# CD206+ tendon resident macrophages and their potential crosstalk with fibroblasts and the ECM during tendon growth and maturation

**DOI:** 10.3389/fphys.2023.1122348

**Published:** 2023-02-22

**Authors:** Catherine A. Bautista, Anjana Srikumar, Elisia D. Tichy, Grace Qian, Xi Jiang, Ling Qin, Foteini Mourkioti, Nathaniel A. Dyment

**Affiliations:** ^1^ McKay Orthopaedic Research Laboratory, Department of Orthopaedic Surgery, University of PA, Philadelphia, PA, United States; ^2^ Department of Bioengineering, School of Engineering and Applied Science, University of PA, Philadelphia, PA, United States; ^3^ Department of Cell and Developmental Biology, Perelman School of Medicine, University of PA, Philadelphia, PA, United States; ^4^ Penn Institute for Regenerative Medicine, Musculoskeletal Program, Perelman School of Medicine, University of PA, Philadelphia, PA, United States

**Keywords:** tendon, fibroblast, development, crosstalk, csf1r signaling, extracellular matrix, growth, resident macrophage

## Abstract

Resident macrophages exist in a variety of tissues, including tendon, and play context-specific roles in their tissue of residence. In this study, we define the spatiotemporal distribution and phenotypic profile of tendon resident macrophages and their crosstalk with neighboring tendon fibroblasts and the extracellular matrix (ECM) during murine tendon development, growth, and homeostasis. Fluorescent imaging of cryosections revealed that F4/80^+^ tendon resident macrophages reside adjacent to Col1a1-CFP^+^ Scx-GFP^+^ fibroblasts within the tendon fascicle from embryonic development (E15.5) into adulthood (P56). Through flow cytometry and qPCR, we found that these tendon resident macrophages express several well-known macrophage markers, including *Adgre1* (F4/80), *Mrc1* (CD206), *Lyve1*, and *Folr2,* but not Ly-6C, and express the Csf1r-EGFP (“MacGreen”) reporter. The proportion of Csf1r-EGFP^+^ resident macrophages in relation to the total cell number increases markedly during early postnatal growth, while the density of macrophages per mm^2^ remains constant during this same time frame. Interestingly, proliferation of resident macrophages is higher than adjacent fibroblasts, which likely contributes to this increase in macrophage proportion. The expression profile of tendon resident macrophages also changes with age, with increased pro-inflammatory and anti-inflammatory cytokine expression in P56 compared to P14 macrophages. In addition, the expression profile of limb tendon resident macrophages diverges from that of tail tendon resident macrophages, suggesting differential phenotypes across anatomically and functionally different tendons. As macrophages are known to communicate with adjacent fibroblasts in other tissues, we conducted ligand-receptor analysis and found potential two-way signaling between tendon fibroblasts and resident macrophages. Tendon fibroblasts express high levels of *Csf1*, which encodes macrophage colony stimulating factor (M-CSF) that acts on the CSF1 receptor (CSF1R) on macrophages. Importantly, *Csf1r*-expressing resident macrophages preferentially localize to *Csf1*-expressing fibroblasts, supporting the “nurturing scaffold” model for tendon macrophage patterning. Lastly, we found that tendon resident macrophages express high levels of ECM-related genes, including *Mrc1* (mannose receptor), *Lyve1* (hyaluronan receptor), *Lair1* (type I collagen receptor), *Ctss* (elastase), and *Mmp13* (collagenase), and internalize DQ Collagen in explant cultures. Overall, our study provides insights into the potential roles of tendon resident macrophages in regulating fibroblast phenotype and the ECM during tendon growth.

## 1 Introduction

Tissue resident macrophages play key roles in the development and function of various tissues (Lee and Ginhoux, 2022). Most resident macrophages found across tissues in adulthood are embryonically derived. The first wave of macrophages originates at murine embryonic day 8.5 (E8.5) from yolk sac-derived erythromyeloid precursors and share a common transcriptional program that diverges with time in their respective tissues of residence (Hoeffel et al., 2015; Mass et al., 2016). The second wave of embryonic macrophages is derived from fetal liver monocytes at E10.5 (Gomez Perdiguero et al., 2015; Bleriot et al., 2020). From approximately E17.5 onwards, bone marrow-derived monocytes give rise to subsets of certain resident macrophage populations (e.g., cardiac resident macrophages, intestinal resident macrophages) as well as macrophages that are recruited to sites of inflammation and injury (Theret et al., 2019; Dick et al., 2022). Resident macrophages influence tissue development and function through secretion of chemokines, growth factors, and other cytokines that act on receptors on neighboring cells (Wynn et al., 2013; Lee and Ginhoux, 2022). Resident macrophages also directly remodel the extracellular matrix (ECM) of various tissues (Wiktor-Jedrzejczak et al., 1990; Pollard, 2009; Harris et al., 2012; Madsen et al., 2013; Wang et al., 2020b; Peck et al., 2022). Their phenotype and function are highly dependent on crosstalk with their local microenvironment, including with neighboring cells and the cytokines and ECM components they secrete.

Certain dense connective tissues, such as tendon, contain resident macrophages but their function is poorly defined during normal growth and development (Harvey et al., 2019; Lehner et al., 2019; De Micheli et al., 2020; Sorkin et al., 2020; Tan et al., 2020; Muscat et al., 2022). The structure of dense connective tissues begets their function and may provide cues that dictate a unique function of macrophages residing in these tissues. Tendon is composed of hierarchically organized, uniaxially aligned type I collagen fibers that function to efficiently transfer loads generated from muscle contraction to bone to drive ambulation of the skeleton. Within the hierarchically organized collagen fascicles exist internal tendon fibroblasts (i.e., tenocytes) situated within linear arrays. The vast majority of these cells within the growing tendon expresses *Scx* and *Col1a1*, and recent studies have highlighted their heterogeneity using single-cell RNA sequencing (scRNA-seq) and various transgenic reporter models (Best and Loiselle, 2019; Harvey et al., 2019; Lehner et al., 2019; De Micheli et al., 2020; Tan et al., 2020; Akbar et al., 2021; Muscat et al., 2022). Interestingly, these scRNA-seq studies suggest that crosstalk between different subpopulations exist, including between fibroblasts and macrophages (De Micheli et al., 2020; Akbar et al., 2021). Nonetheless, we have a limited understanding of these different cell populations within tendon and how they participate together to orchestrate development, growth, and homeostasis of this unique tissue.

Tendon growth is driven first by a highly proliferative phase that begins during embryonic development and transitions shortly after birth (i.e., ∼2–3 weeks in a mouse) to a phase with rapid ECM synthesis and maturation (Ansorge et al., 2011; Grinstein et al., 2019). While the majority of the tendon ECM is conserved through adulthood, a subset of collagen is remodeled on a daily basis to maintain homeostasis (Heinemeier et al., 2013; Chang et al., 2020; Zhang et al., 2020). Furthermore, there are several non-collagenous components of the tendon ECM that exhibit high turnover rates (Thorpe et al., 2010; Choi et al., 2020). While resident macrophages have known roles in ECM remodeling during the growth and development of several tissues (Wiktor-Jedrzejczak et al., 1990; Ingman et al., 2006; Pollard, 2009; Harris et al., 2012; Madsen et al., 2013; Wang et al., 2020b; Peck et al., 2022), we know very little about their role in the growth and homeostasis of tendon.

In this study, we characterized the proliferation dynamics, localization, and phenotype of tendon resident macrophages at multiple stages of murine development and growth. We uncovered evidence of crosstalk between tendon resident macrophages and tendon fibroblasts and found that resident macrophages may be important regulators of the ECM. Our results provide new insights into the cellular mechanisms that regulate postnatal tendon development and growth.

## 2 Materials and methods

### 2.1 Mouse models

All animal housing, care, and experiments were performed in accordance with the University of Pennsylvania Institutional Animal Care and Use Committee. The genetic constructs used within mice in this study were described previously: 1) a 3.6 KB fragment of the Col1a1 promoter driving the expression of the CFP reporter (Tg(Col1a1*3.6-Cyan)2Rowe/J; “Col1CFP”) (Kalajzic et al., 2002), 2) a Scx promoter driving the expression of the GFP reporter (Tg(Scx-GFP)1Stzr; “ScxGFP”) (Pryce et al., 2007), 3) a Scx promoter driving the expression of Cre recombinase (“ScxCre”) (Blitz et al., 2013), 4) a tdTomato reporter driven by Cre-mediated recombination (B6; 129S6-Gt (ROSA) 26Sortm9 (CAG-tdTomato) Hze/J; “Ai9”) (Madisen et al., 2010), and 5) a Csf1r promoter driving the expression of the EGFP reporter (B6.Cg-Tg(Csf1r-EGFP)1Hume/J; “Csf1rGFP”) (Sasmono et al., 2003). The Col1CFP and ScxGFP lines were crossed to obtain Col1CFP;ScxGFP double transgenic mice used for F4/80 immunofluorescence (IF). The Col1CFP line was used for F4/80 IF and explant culture. The ScxCre and Ai9 lines were crossed to obtain ScxCre;Ai9 tenogenic lineage reporter mice for F4/80 IF and fluorescence-assisted cell sorting (FACS) for gene expression studies. Csf1rGFP;ScxCre;Ai9 mice were generated and used for cryohistology. Csf1rGFP reporter mice were used for macrophage abundance, Euclidean distance mapping, flow cytometry (FC), and cell proliferation studies. Wild-type CD-1 IGS mice (Charles River Strain 022; “CD1”) were used for FC and *in situ* hybridization (ISH).

### 2.2 EdU labeling

For cell proliferation analysis, P1 animals were weighed and injected with 6 μg/g 5-ethynyl-2ʹ-deoxyuridine (EdU) 4 h prior to sacrifice (n = 4 animals). Knees were fixed, sectioned (see “Tissue harvest and sectioning for cryohistology” section), and stained with the Click-&-Go Cell Reaction Buffer Kit (Click Chemistry Tools Cat. No. 1263) and Alexa Fluor 647 Azide (Invitrogen Cat. No. A10277). Stained sections were then coverslipped and imaged (see “Fluorescent imaging” section) and quantified (see “Fluorescent image analysis” section).

### 2.3 Tissue harvest and sectioning for cryohistology

Prior to fixation, animals were euthanized, skin was removed, and hindlimbs were cut from the body. For F4/80 immunofluorescence (IF) and reporter imaging, hindlimbs were fixed in 4% (v/v) phosphate-buffered paraformaldehyde (PFA) solution (Electron Microscopy Sciences 15714-S) for 3 h on an orbital shaker and incubated in PBS overnight at 4°C (*n* = 3 to 4 animals per line per time point). For EdU staining, hindlimbs were fixed in 10% neutral buffered formalin (Azer Scientific CUNBF-5-G) overnight at 4°C and incubated in 30% (w/v) sucrose (Sigma-Aldrich S8501) overnight at 4°C (*n* = 4 animals). For RNAScope *in situ* hybridization (ISH), hindlimbs were fixed in 4% PFA overnight at 4°C and incubated in 30% sucrose overnight at 4°C (*n* = 3 animals). Fixed knee and ankle joints were embedded and frozen in optimal cutting temperature (OCT) compound. 8-µm frozen sagittal sections were collected using a previously established tape stabilization procedure (Dyment et al., 2016). Sections on Cryofilm (Section-Lab) were adhered to glass slides using a 0.75% (w/v) chitosan (Sigma-Aldrich 419419) in 0.25% (v/v) acetic acid (Sigma-Aldrich 695092) prior to staining.

### 2.4 Immunofluorescence staining

For immunofluorescence (IF) experiments, patellar tendon sections were rinsed in PBS to remove OCT compound, incubated with 20 μg/mL Proteinase K for 10 min at room temperature (RT) for antigen retrieval, and stained with rat anti-F4/80 primary antibody (clone BM8; Biolegend 123102) in 5% goat serum (Sigma-Aldrich G9023) in PBS overnight at 4°C. Sections were then rinsed with PBS and stained with goat anti-rat Alexa Fluor 555 (for Col1CFP;ScxGFP sections) or Alexa Fluor 647 (for ScxCre;Ai9 sections) secondary antibody (Invitrogen A21434; Invitrogen A21247) for 1 h at RT.

### 2.5 Fluorescent imaging

IF, fluorescent reporter-only, and EdU-stained sections were rinsed with PBS and mounted with 30% (v/v) glycerol (Invitrogen 15514011) containing Hoechst 33342 (Thermo Scientific 62249) to label nuclei prior to imaging. Whole sections were imaged on the Zeiss Axio Scan.Z1 with the N-Achroplan 20X/0.45 PolM27 objective and Colibri.7 LED.

### 2.6 Fluorescent image analysis

Fiji software was used for all fluorescent image analysis and all data processing and plotting was done using custom R scripts in the RStudio integrated development environment using the “plyr” and “tidyverse” packages. First, the selection tool was used to manually segment the tendon fascicle from surrounding tissue in Fiji. Intensity thresholding was applied to the Hoechst (nuclei)-only channel and watershed segmentation was applied to the resulting binary images to obtain individual nuclei. The “Analyze Particles” function was used to quantify the number of total nuclei in each section. For the F4/80 IF analysis, F4/80^+^ cells were counted manually (three to four sections per sample). For the Csf1rGFP analysis, the “Analyze Particles” function was used to quantify the mean GFP intensity within each nuclear mask (three to four sections per sample). For the cell proliferation analysis, GFP^+^ and EdU^+^ cells were counted manually due to the high cellularity of P1 tendons (9-14 sections per sample).

### 2.7 Euclidean distance map analysis

Fiji software was used to quantify the shortest distance of each cell to the tendon surface for the Csf1rGFP patellar tendon image set. Images of sections with major sectioning artifacts were excluded. Segmented lines were drawn along the anterior and posterior surfaces of the patellar tendon and a 16-bit Euclidean distance map (EDM) was generated, where the pixel intensity was zero at the posterior and anterior surfaces and increased as distance from the surfaces (depth) increased. We generated nuclear masks as previously described (see “Fluorescent image analysis” section) and used the “Analyze Particles” function to count the number of cells and to measure the mean intensity of the GFP and EDM channels. We applied a standard threshold across all images to define GFP^+^ and GFP^–^ cells. For each image, we normalized each EDM value by the maximum EDM value (EDM_max_) and divided the range by four to define four increments of depth. Quartile 1 (“Q1”) represents the outermost increment and Quartile 4 (“Q4”) represents the innermost increment. We binned each cell into one of the four quartiles and counted the number of GFP^+^ and GFP^–^cells within each quartile to determine the percentage of each population as a function of depth. To exclude the effects of differences in overall percentages of each population due to age, we normalized the percentage of cells within each quartile by the percentage of cells in the whole section. All data processing and plotting was performed using RStudio using the “plyr”, “tidyverse”, and “reshape2” packages.

### 2.8 Analysis of single-cell RNA-sequencing datasets

Publicly available single-cell RNA sequencing (scRNA-seq) datasets for P7 limb tendons and 3-month-old patellar tendons were obtained from the NCBI under GSE139558 (Tan et al., 2020) and PRJNA506218 (Harvey et al., 2019), respectively. Count matrices were filtered, normalized, and scaled using Seurat v3.1 (Stuart et al., 2019). Principal component analysis (PCA) was performed, cells were clustered using graph-based clustering, and UMAP non-linear dimensional reduction was performed. Differentially expressed genes were identified using the Seurat “FindMarkers” function. The Seurat function “FeaturePlot” was used to visualize individual gene expression on UMAP plots.

### 2.9 Preparation of cell suspensions for flow cytometry and fluorescence-activated cell sorting

Three animals were pooled for each sample. Following euthanasia, limbs were skinned, cut from the body, and placed in DMEM (Gibco 11965084) on ice. Muscle, bone, fat and surrounding peritenon tissue were grossly dissected from limb tendons prior to being cut from the body. For flow cytometry (FC), all tendons within each hindlimb were pooled together; the larger tendons (patellar, Achilles, and Flexor digitorum longus tendons) were cut into smaller 1–2-mm pieces. 1-cm segments were cut from the tails and 30-40 tail tendon fascicles were isolated from each segment. Macrophage marker FC was performed on hindlimb and tail tendon cells from P14 and P56 CD1 animals across three independent experiments per time point. For Csf1rGFP F4/80 FC validation, hindlimb and tail tendon cells from P28-P35 Csf1rGFP animals were analyzed across three independent experiments. For fluorescence-activated cell sorting (FACS) and gene expression studies, forelimb tendons were pooled along with the hindlimb tendons. FACS was performed on limb and tail tendon cells from P14 and P56 ScxCre;Ai9 animals (*n* = 5 samples per tissue per time point).

Dissected tendons were digested in 4 mg/mL Type 4 Collagenase (Worthington LS004189) 3 mg/mL Dispase II (Sigma-Aldrich D4693) in DMEM containing 10 mM HEPES and 2% Penicillin/Streptomycin/Fungizone on an orbital shaker at 37°C for 0.5–2.5 h, depending on tendon and age, until tendons were mostly digested and translucent. Cells were resuspended in DMEM containing 10% FBS and passed through a 70-µm strainer, then resuspended in Flow Buffer (HBSS containing 1% (w/v) BSA and 25 mM HEPES) and passed through a 30-µm strainer prior to staining.

For macrophage marker FC, cells were stained with anti-F4/80-Brilliant Violet 421 (clone BM8; Biolegend 123131), anti-CD11b-Brilliant Violet 785 (clone M1/70; Biolegend 101243), anti-Ly6C-FITC (clone HK1.4; Biolegend 128005), anti-CD206-PE (clone C068C2; Biolegend 141705), anti-CD86-Alexa Fluor 647 (clone GL-1; Biolegend 105020), and LIVE/DEAD Fixable Near IR (Invitrogen L10119) in PBS for 15 min at RT. For Csf1rGFP F4/80 FC validation, cells were stained with anti-F4/80-Brilliant Violet 421, anti-CD11b-Brilliant Violet 785, and LIVE/DEAD Fixable Near IR. Stained cells were rinsed with Flow Buffer, fixed in 4% PFA for 15 min at 4°C, resuspended in Flow Buffer, and strained prior to analysis on the BD LSRFortessa at the Penn Cytomics and Cell Sorting Resource Laboratory. FC data were analyzed using FlowJo, R, and RStudio.

For FACS, cells were stained with F4/80-Brilliant Violet 421 and LIVE/DEAD Fixable Near IR in PBS for 15 min at RT. Cells were rinsed with Flow Buffer and sorted on the BD FACSAria II into Flow Buffer to obtain Brilliant Violet 421^+^ tdTomato^–^ cells (macrophages) and Brilliant Violet 421^–^ tdTomato^+^ cells (fibroblasts). Sorted cells were immediately processed for RNA extraction. A total of 40 cell samples (2 populations per tissue per time point) were collected for gene expression analysis.

### 2.10 Preparation of cDNA for gene expression analysis

Sorted cells were resuspended in TRIzol LS (Invitrogen 10296010) and RNA was extracted using the Direct-zol RNA Microprep kit (Zymo Research R2062). cDNA was synthesized and pre-amplified as described previously (Tsinman et al., 2021). Briefly, isolated RNA was reverse transcribed into cDNA using the SuperScript IV VILO Master Mix with ezDNase Enzyme (Invitrogen 11766050). cDNA was pre-amplified for 15 cycles using the Preamp Master Mix (Fluidigm 100–5580) with a pool of all TaqMan Gene Expression Assays (Applied Biosystems 4351372), except those for the highly expressed genes *18s* and *Col1a1* (see Supplementary Table S1 for list of all genes and corresponding TaqMan Assay ID numbers).

### 2.11 High-throughput qPCR

qPCR of sorted macrophage and fibroblast samples was performed on the Fluidigm Biomark HD platform at the Penn Molecular Profiling Facility using a 96.96 Dynamic Array IFC (Fluidigm BMK-M-96.96) loaded with 40 pre-amplified cDNA samples in duplicate and 96 20 x TaqMan Gene Expression Assays (see Supplementary Table S1 for TaqMan Assay ID numbers). The mean C_T_ value across technical duplicates was calculated for each reaction. Sample measurements for *Il2* and *Il2ra* were excluded because multiple samples within both the macrophage and fibroblast populations had undetectable measurements. Sample measurements for *Sema4c* were also excluded due to abnormal amplification curves across samples. ∆C_T_ values were used to compare gene expression across samples. To calculate ∆C_T_, for each sample, the C_T_ value for each gene of interest was subtracted from the average C_T_ value of the three housekeeping genes (*18s*, *Abl1*, and *Rps17*). Log_2_(Fold Change) for P56 limb vs P14 limb and for P56 limb vs P56 tail was determined by computing ∆∆C_T_^limb^ = ∆C_T_^P56 limb^—∆C_T_^P14 limb^ and ∆∆C_T_^P56^ = ∆C_T_^P56 limb^—∆C_T_^P56 tail^, respectively. All data were analyzed and plotted using R and RStudio with the “tidyverse”, “reshape2”, and “ggrepel” packages.

### 2.12 Principal component analysis

For principal component analysis (PCA), hierarchical clustering, and heatmap generation, ∆C_T_ values for all genes (excluding the housekeeping genes) were uploaded into ClustVis (Metsalu and Vilo, 2015). For PCA and hierarchical clustering of just the macrophage samples, only measurements for the genes with a global PC1 loading value greater than zero were entered into ClustVis. All plotting was done using custom R scripts using the “ggplot2” package. All pre- processing parameters, as well as the global principal component analysis (PCA) of the Fluidigm qPCR dataset are saved in ClustVis under the settings ID: YDoRZCkjkjocViB. For the analysis of just the macrophage samples, all pre-processing parameters and PCA outputs are saved in ClustVis under the settings ID: tTPjCrJlxkZjpad.

### 2.13 RNAScope *in situ* hybridization and analysis

*In situ* hybridization (ISH) was performed using the RNAScope 2.5 HD Duplex Assay (ACD 322430) (Koyama et al., 2021). The Mm-Csf1r-C1 and Mm-Csf1-C2 probes (ACD 428191; ACD 315621-C2) were used to visualize the expression of *Csf1r* and *Csf1*, respectively, in P28 knee sections. All procedures were performed according to manufacturer’s instructions, except the Target Retrieval steps were excluded and a custom protease (ACD 300040) was used instead of the standard proteases. Additionally, to minimize color overlap during quantification, Hoechst was used instead of hematoxylin to stain nuclei. Sections with only Csf1r-C1 (green), only Csf1-C2 (red), and only Hoechst were included as single-color controls to determine baselines for color deconvolution. Control sections were also prepared using the Positive Control Probe (ACD 320881) and Negative Control Probe (ACD 320751) to ensure sensitivity and specificity of the assay. Stained sections were imaged on the Zeiss Axio Scan.Z1.

ISH images were processed using the Fiji Colour Deconvolution plugin to obtain separate channels for green (Csf1r-C1), red (Csf1-C2), and background colors. Separated images were inverted such that brighter signal indicated stronger staining. Intensity per 50 × 50-µm^2^ unit area within the tendon region of interest was quantified for each channel (green, red, and Hoechst) using the “Analyze particles” function.

### 2.14 Collagen internalization explant culture

Tail tendon fascicles were isolated from two P35 Col1CFP mice (*n* = 5 tendons per animal) and cultured with DMEM containing 2% FBS, 1% PSF, and 10 μg/mL DQ Collagen, type I From Bovine Skin, Fluorescein Conjugate (Invitrogen D12060) at 37°C, 5% CO_2_. Culture media was exchanged daily. Live tail tendons were stained with Hoechst and imaged on the Zeiss Axio Scan.Z1 to visualize localization of Hoechst, Col1CFP, and fluorescein after 2 days. After 3 days, tail tendons were cut from the slides, pooled, and digested with 4 mg/mL Type 4 Collagenase and 3 mg/mL Dispase II on an orbital shaker for 1 h at 37°C to obtain a single-cell suspension. Cells were stained with anti-CD206-PE, anti-F4/80-Alexa Fluor 647 (Biolegend 123121), and LIVE/DEAD Fixable Near IR in PBS for 30 min at 4°C. Stained cells were then rinsed and resuspended in Flow Buffer transferred to chamber slides (Invitrogen C10228) and imaged on the Zeiss Axio Scan.Z1. In Fiji, nuclear masks were generated and mean intensity for each channel was calculated as before (see “Fluorescent image analysis” section). Plots were generated using the R “tidyverse” packages in RStudio.

### 2.15 Statistical analysis

The Shapiro-Wilk test was used to assess normality for comparisons of percentages of F4/80^+^ cells, flow cytometry percentages, percentages of GFP^+^ cells, cell densities, percentages of EdU^+^ cells, EDM ratios, and ∆C_T_. An F test was used to test for equal variances for comparisons of flow cytometry percentages, percentages of EdU^+^ cells, EDM ratios, and ∆C_T_. Levene’s test was used to test for homogeneity of variances for comparisons of percentages of F4/80^+^ cells, percentages of GFP^+^ cells, and cell densities using the R “car” package. Percentages of F4/80^+^ cells, percentages of GFP^+^ cells, cell density measurements, and EDM ratios were compared *via* one-way ANOVA with Tukey’s *post hoc* test. Flow cytometry percentages, global PC1 scores, ∆C_T_, and ∆∆C_T_ were compared using the Mann-Whitney U test. Percentages of EdU^+^ cells were compared *via* paired *t*-test. Spearman’s rank correlation coefficient ρ was calculated for RNAScope ISH intensity per unit area correlations. All tests were performed using custom R scripts and with the significance level α was set to 0.05.

## 3 Results

### 3.1 F4/80^+^ Csf1rGFP^+^ macrophages reside adjacent to tendon fibroblasts within the tendon fascicle during embryonic development and postnatal growth

**FIGURE 1:**
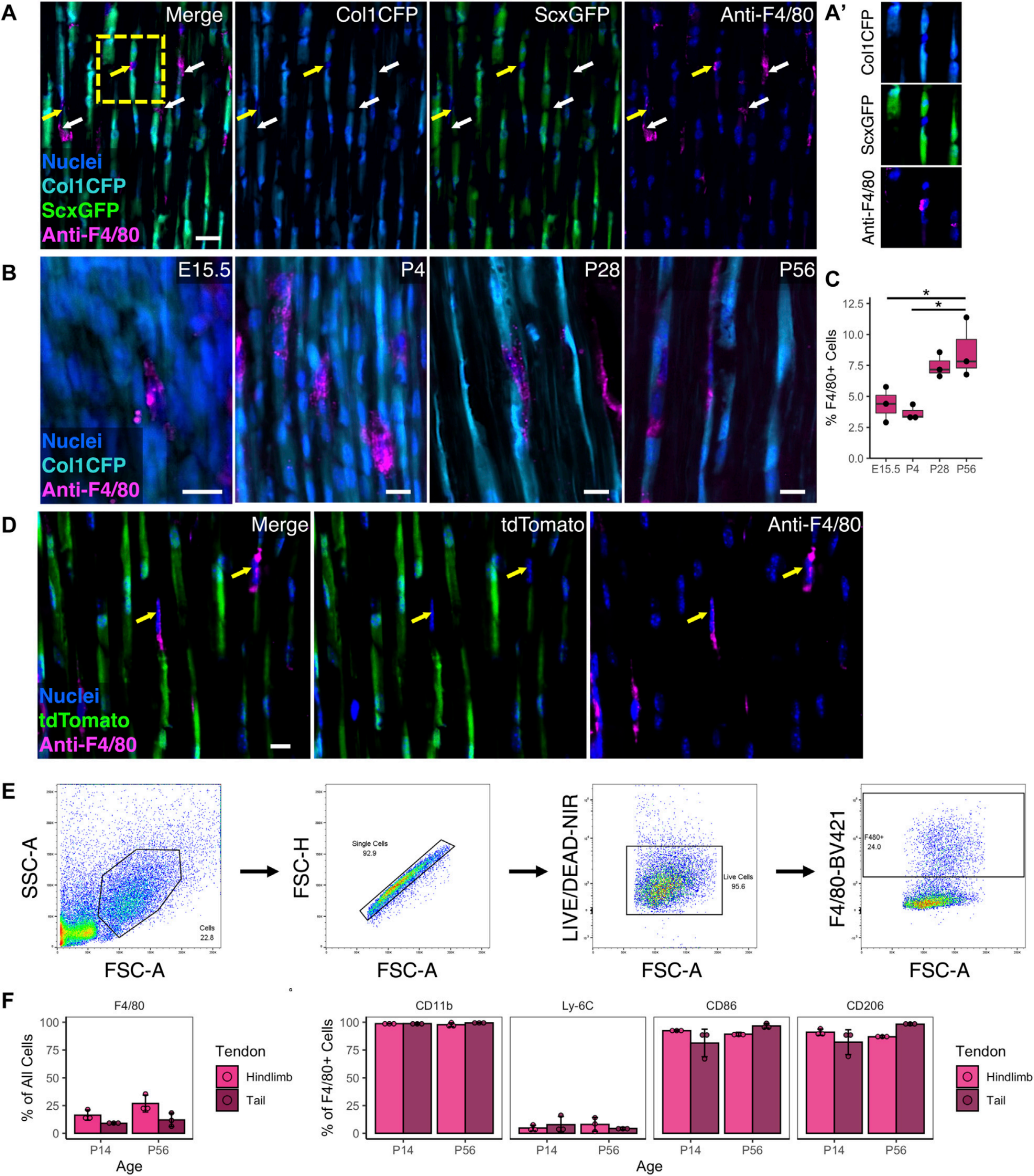
Resident macrophages are positioned adjacent to fibroblasts during tendon development and postnatal growth **(A)** Representative image of P28 Col1CFP;ScxGFP patellar tendon section immunostained for pan-macrophage marker F4/80 at P28 showing that Col1CFP^−^ ScxGFP^–^ cells are F4/80^+^ macrophages. Yellow arrows indicate F4/80^+^ macrophages with nuclei in plane; white arrows indicate F4/80^+^ cytoplasmic projections **(A')** Inset of F4/80^+^ macrophage within linear array of fibroblasts. Scale bar = 20 µm **(B)** Representative images of E15.5, P4, P28, and P56 Col1CFP patellar tendon sections immunostained for F4/80. Scale bar = 10 µm **(C)** Quantification of F4/80^+^ cells in patellar tendon sections. Data shown as mean ± SD (*n* = 3). *p < 0.05 as determined by one-way ANOVA with Tukey’s post-hoc test **(D)** F4/80 immunostaining on P28 ScxCre;Ai9 patellar tendon sections demonstrating that F4/80^+^ cells are not tdTomato^+^. Scale bar = 10 µm **(E)** Representative images of gating strategy for surface marker characterization of tendon resident macrophages by flow cytometry **(F)** Percentage of F4/80^+^ cells in hindlimb and tail tendons at P14 and P56 **(G)** Percentage of marker expression within the F4/80^+^ population. Data shown as mean ± SD (*n* = 3). There were no significant differences (*p* > 0.05) between tendons or between ages as determined by Mann Whitney U test.

Resident macrophages exist in numerous tissues and organs in the body and are derived from either embryonic (e.g., yolk sac) or adult origins. While macrophages are critical to tendon healing, their spatial distribution and role during normal growth and development is unknown. To establish the presence of tendon resident macrophages and their localization in relation to resident tendon fibroblasts, we performed immunofluorescence (IF) staining for the murine pan-macrophage marker F4/80 in mice expressing the tendon fibroblast markers Col1a1(3.6 kb)-CFP (Col1CFP) and Scleraxis-GFP (ScxGFP). We found that F4/80^+^ resident macrophages reside adjacent to Col1CFP^+^ ScxGFP^+^ tendon fibroblasts throughout growth and development (Figure 1A). They are present within the tendon midsubstance as early as embryonic day 15.5 (E15.5), right after tendon formation, and increased during postnatal growth into early adulthood (P4, P28, and P56) (Figures 1B, C).

F4/80 IF revealed that tendon resident macrophages did not express the tenocyte reporters Col1CFP and ScxGFP at any age (Figures 1A, B). In tendons from ScxCre;Ai9 mice, where cells from a tenogenic origin are tdTomato^+^, we found that F4/80^+^ resident macrophages did not express tdTomato (Figure 1D). Flow cytometry (FC) analysis indicated that F4/80^+^ resident macrophages co-express CD11b (a myeloid marker), CD86 (which is expressed by activated B and T cells, monocytes, macrophages, dendritic cells, and astrocytes), and CD206 (which is expressed by macrophages, dendritic cells, Langerhans cells, and hepatic or lymphatic endothelial cells) (Figures 1E–G; Supplementary Figure S1). Due to the difficulty in immunostaining F4/80 in tendon sections, we utilized the MacGreen mice that express Csf1r-EGFP (Csf1rGFP), which labels resident macrophages in various tissues throughout the body (Sasmono et al., 2003), to conduct a rigorous spatiotemporal analysis. In tendons, Csf1rGFP labeled nearly all CD11b^+^ F4/80^+^ cells (98.1 ± 0.8% in hindlimb tendons and 99.0 ± 0.3% in tail tendons; Supplementary Figure S2A). In tendons from Csf1rGFP;ScxCre;Ai9 mice, nearly all Csf1rGFP^–^ cells were positive for tdTomato (Supplementary Figure S2B). Therefore, we classified Csf1rGFP^–^ cells within the tendon fascicle as scleraxis-lineage cells (i.e., tendon fibroblasts) in subsequent experiments. Csf1rGFP^+^ cells were not present within the midsubstance of the ligaments investigated (anterior and posterior cruciate ligaments) or the entheses within either tendons or ligaments (Supplementary Figure S3). The spatial distribution of macrophages was uniform along the length of the patellar tendon midsubstance. In contrast, the density of macrophages in the Achilles tendon appeared to increase from the distal region near the calcaneus to the proximal region near the myotendinous junction (Supplementary Figure S3B).

Tendon resident macrophages had diverse morphologies, as shown in cryosections from P28 Csf1rGFP;ScxCre;Ai9 patellar tendons (Supplementary Figure S4). We found macrophages in three general morphological states: 1) macrophages with nuclear and cell body shapes similar to fibroblasts, 2) elongated macrophages with high aspect ratios (major axis divided by minor axis), and 3) macrophages with extensions that wrap around the cell bodies of neighboring tdTomato^+^ fibroblasts. This close association of macrophages and tendon fibroblasts within the linear arrays in the tendon midsubstance indicates potential cell-cell communication between these different cell populations.

### 3.2 The proportion of resident macrophages within the tendon midsubstance increases during postnatal growth

**FIGURE 2:**
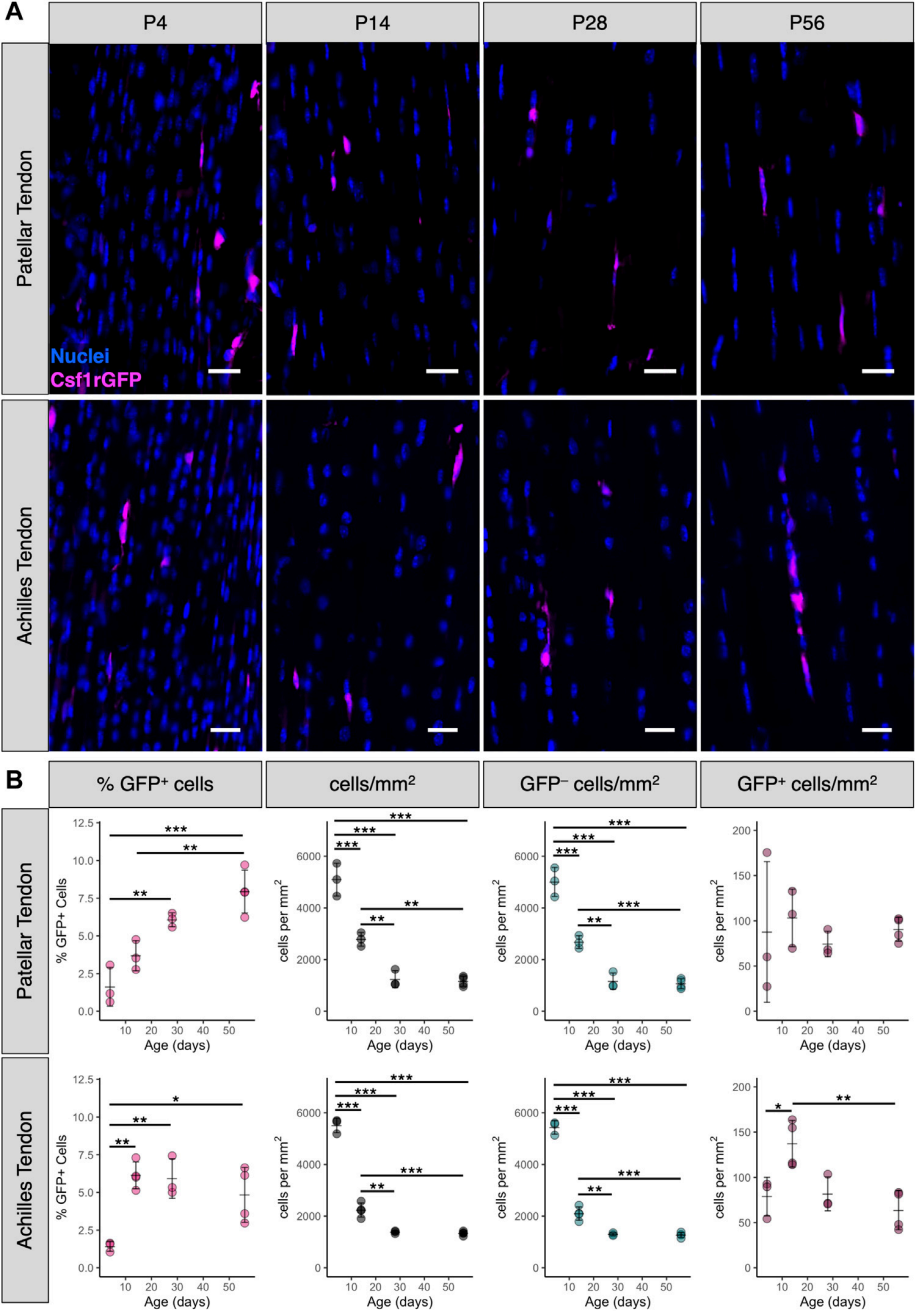
Percentage of tendon resident macrophages increases during growth in patellar and Achilles tendons **(A)** Representative images of Csf1rGFP patellar and Achilles tendon sections at P4, P14, P28, and P56. Scale bar = 20 µm **(B)** Quantification of Csf1rGFP^+^ cells in patellar and Achilles tendon sections. Data shown as mean ± SD (*n* = 3–4). *p < 0.05, **p < 0.01, ***p < 0.001 as determined by one-way ANOVA with Tukey’s post-hoc test.

During postnatal growth, tendon length, cross-sectional area, and total cell number increase dramatically (Ansorge et al., 2011; Grinstein et al., 2019). To measure the abundance and distribution of tendon resident macrophages during this time period, we quantified sagittal cryosections of patellar and Achilles tendons from P4, P14, P28, and P56 Csf1rGFP mice (Figure 2A). While there were numerous Csf1rGFP^+^ cells in the peritenon surrounding the tendon (Supplementary Figure S3), we excluded this region from our quantification in order to focus on the macrophage population within the tendon fascicle. In the patellar tendon, the mean percentage of Csf1rGFP^+^ cells significantly increased from 1.6 ± 1.3% at P4 to 7.9 ± 1.4% at P56 (Figure 2B; *p* < 0.001). In the Achilles tendon, the mean percentage of Csf1rGFP^+^ cells increased from 1.4 ± 0.3% at P4 to 4.8 ± 1.8% at P56 (*p* < 0.05). As expected, the total cell density (cells/mm^2^) and the fibroblast density (GFP^–^ cells/mm^2^) significantly decreased during this period of matrix synthesis-driven postnatal growth (Figure 2B; *p* < 0.001). While the proportion of macrophages increased during growth, the spatial density of macrophages (GFP^+^ cells/mm^2^) remained relatively constant over the same period, suggesting there is a certain stimulus that maintains this spatial density.

### 3.3 Tendon resident macrophages proliferate within the neonatal tendon at a higher rate than fibroblasts

**FIGURE 3:**
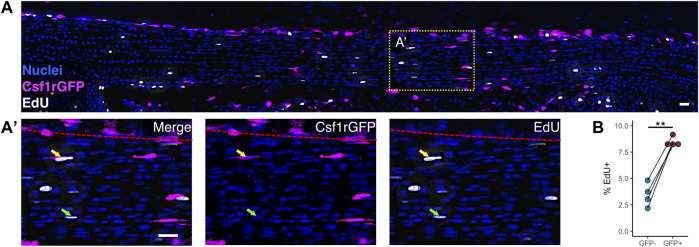
Macrophages proliferate at a higher rate than fibroblasts within the neonatal tendon **(A)** Representative image of EdU staining of proliferating cells in P1 Csf1rGFP patellar tendon sections. Green arrows indicate EdU^+^ Csf1rGFP^–^ cells; yellow arrows indicate EdU^+^ Csf1rGFP^+^ cells; red dotted lines indicate border between tendon fascicle and peritenon. Scale bar = 20 µm **(B)** Quantification of EdU^+^ cells within Csf1rGFP^–^ fibroblast and Csf1rGFP^+^ macrophage populations (*n* = 4). **p < 0.01 as determined by paired *t*-test.

Early postnatal tendon growth (P0-P14) is characterized by a high degree of proliferation of the overall tendon cell population in addition to increases in total tendon volume (Ansorge et al., 2011; Liu et al., 2012; Grinstein et al., 2019). Using the H2B-GFP mouse model to assess proliferation rates in the Achilles tendon, Grinstein et al. found that, on average, 19% of tendon cells divide per day from P0 to P7. Because proliferation is the main driver of changes in cell number during tendon growth, we measured the proliferation rates of macrophages and fibroblasts at P1 to determine if differences in proliferation rate drive the increase in macrophage proportion that we observed during neonatal growth. We chose P1 instead of later time points because this is approximately the age when the percentage of proliferating cells peaks postnatally in the tendon (Liu et al., 2012; Grinstein et al., 2019), allowing us to count enough proliferating cells per population per tendon to perform a detailed analysis. To test whether macrophages proliferated at a higher rate than fibroblasts in the neonatal tendon, we injected P1 Csf1rGFP mice with EdU 4 h prior to sacrifice to label proliferating cells. We then stained patellar tendon cryosections with a fluorescent azide to visualize EdU colocalization with GFP (Figure 3A). We found that Csf1rGFP^+^ macrophages residing within the tendon fascicle proliferated at a 2.5 times greater rate than Csf1rGFP^–^ tendon fibroblasts (Figure 3B; *p* = 0.003). This suggests that the increase in proportion of macrophages during early postnatal growth is driven by a higher proliferation rate compared to fibroblasts.

If the increase in proportion of macrophages during early postnatal growth was due to an influx of macrophages or macrophage precursors from outside the tendon, we would expect that the density of macrophages would be higher near the surface of the tendon to correspond with these cells infiltrating the tendon. Using Euclidean distance mapping, we quantified the distance of each Csf1rGFP^+^ macrophage from the tendon surface and compared the distances to those of CsfrGFP^–^ fibroblasts (Supplementary Figure S5A). We normalized the numbers of macrophages and fibroblasts at a certain distance away from the tendon surface by dividing by the total cell number at that same distance (Supplementary Figure S5B). We conducted this normalization to account for differences in tendon thickness that occur along the length of each sagittal section. Using this analysis, we found that macrophages, similar to fibroblasts, were uniformly distributed across the depth of the tendon throughout postnatal growth (Supplementary Figures S5C,D; *p* > 0.05). Taken together, these data suggest that the ratio of macrophages to fibroblasts increases during tendon growth due to a higher rate of proliferation rather than infiltration of extrinsic macrophage precursors.

### 3.4 Spatiotemporal gene expression profile of tendon resident macrophages

**FIGURE 4:**
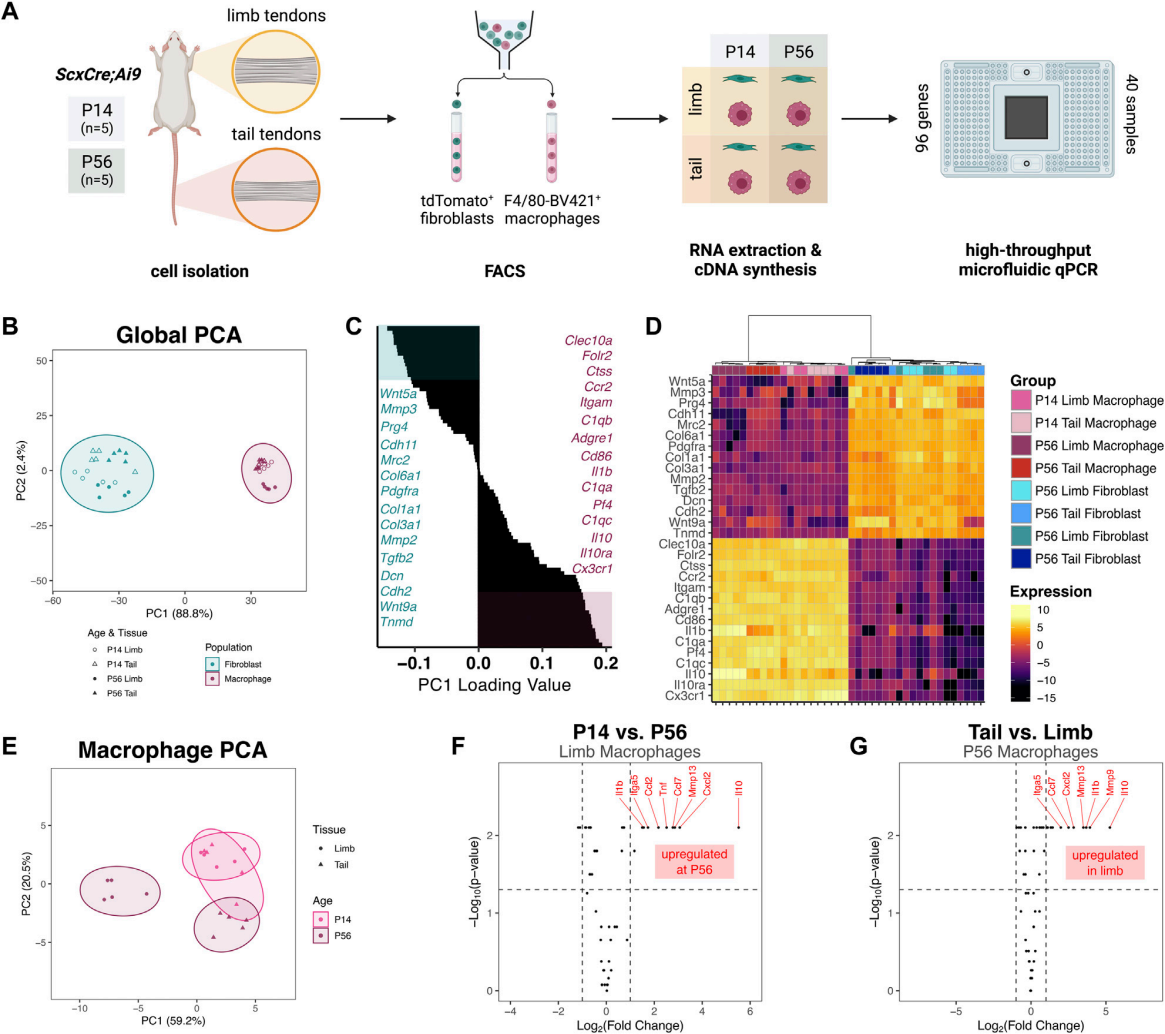
Gene expression profiles of tendon resident macrophages varies by source tendon and age **(A)** Experimental design for FACS and gene expression assay. Cells were isolated from limb tendons and tail tendons collected from P14 and P56 ScxCre;Ai9 mice (*n* = 5 samples per tissue per time point). Cell suspensions were stained with anti-F4/80-Brilliant Violet 421 and subjected to FACS to obtain tdTomato^+^ fibroblasts and F4/80-Brilliant Violet 421^+^ (F4/80-BV421^+^) macrophages. RNA was extracted from each of 40 sorted cell suspensions and converted to cDNA. The 40 unique cDNA samples were preamplified then loaded in duplicate (80 samples total) onto a Fluidigm microfluidic qPCR chip along with 96 TaqMan Gene Expression Assays **(B)** PC1 score vs PC2 score from PCA of all samples and genes. Prediction ellipses represent 95% confidence intervals **(C)** Waterfall plot of PC1 loading values with top 15 (teal) and bottom 15 (maroon) genes highlighted **(D)** Heatmap of genes with highest 15 and lowest 15 PC1 loading values with hierarchical clustering. Genes with PC1 loading values >0 were selected for subsequent macrophage-only PCA **(E)** PC1 score vs PC2 score from PCA of macrophage groups only **(F)** Volcano plot comparing P56 limb tendon macrophage vs P14 limb tendon macrophage gene expression. Horizontal dotted line indicates *p* = 0.05 threshold; left vertical dotted line indicates fold change of −1; right vertical line indicates fold change of 1 **(G)** Volcano plot comparing P56 limb tendon macrophage vs P56 tail tendon macrophage gene expression.

To begin to elucidate the potential roles of resident macrophages during postnatal tendon growth, we measured the gene expression profile of sorted resident macrophages and tendon fibroblasts from different anatomical sites with distinct embryonic origins. We isolated cells from limb and tail tendons from P14 and P56 ScxCre; R26R-tdTomato mice, sorted for tdTomato^+^ F4/80^–^ fibroblasts and tdTomato^–^ F4/80^+^ macrophages, and extracted RNA from each population (Figure 4A). In order to obtain enough RNA from the lowly abundant resident macrophages for each sample, we pooled all limb tendons together. We assessed the expression of 96 genes using the Fluidigm Biomark HD platform. The genes analyzed were selected based on known fibroblast and macrophage markers, cell-cell signaling markers, and cell-ECM markers. Tendon-specific macrophage markers and cell-cell signaling genes were selected based markers obtained after re-analyzing publicly available single-cell RNA-sequencing (scRNA-seq) datasets (Supplementary Figure S6) (Harvey et al., 2019; De Micheli et al., 2020; Tan et al., 2020). Three genes (*18s*, *Abl1*, *Rp17*) were used as housekeeping genes and three genes (*Il2*, *Il2ra*, *Sema4c*) were excluded for technical reasons.

The relative expression levels of each gene for all samples are displayed in Supplementary Figure S7. Principal component analysis (PCA) revealed that the first principal component (PC) accounted for 88.8% of the total variance across tissue, population, and age (Figure 4B). Mapping of PC1 vs PC2 revealed that PC1 separated macrophages from fibroblasts, with the macrophage populations having a significantly higher PC1 score than the fibroblast populations (*p* < 0.001). Genes with some of the highest PC1 loading values (*Cx3cr1*, *C1qc*, *Adgre1*) were genes classically associated with immune cells, whereas genes with some of the lowest PC1 loading values (*Prg4*, *Col1a1*, *Tnmd*) were associated with tendon fibroblasts (Figures 4C, D). Hierarchical clustering also confirmed the separation of fibroblasts from macrophages by the genes surveyed (Figure 4D). Altogether, these findings confirm that fibroblasts and macrophages were properly isolated from the tendons and identify tendon-specific macrophage markers. Within the macrophage population, P56 limb tendon macrophages clustered farther away from the three other macrophage populations. The P14 tail tendon macrophages clustered more closely to the P14 limb tendon macrophages than to the P56 tail tendon macrophages, suggesting the presence of a common early macrophage phenotype that diverges over the course of growth.

To further investigate the divergence in expression within the macrophage population, we performed PCA on only the macrophage samples using a subset of 50 genes whose global PC1 loading values were greater than zero (i.e., more abundant in macrophages than fibroblasts). The first two PCs accounted for 79.7% of the total variance across tissue and age. Mapping of PC1 vs PC2 confirmed that P56 limb tendon macrophages diverged from the other macrophage subpopulations, which had higher PC1 scores (Figure 4E). Within the limb tendons, genes significantly upregulated in P56 macrophages compared to P14 macrophages included the cytokines *Il10*, *Cxcl2*, *Ccl7*, *Tnf*, and *Ccl2* and the catabolic enzyme *Mmp13* (Figure 4F). Box plots of the expression levels of the eight most highly upregulated genes are displayed in Supplementary Figure S8A. Out of 50 genes investigated, 38 genes (76%) were not differentially expressed in limb tendon macrophages at P14 compared to P56. Within the P56 tendons, genes upregulated in limb macrophages compared to tail tendon macrophages included the cytokines *Il10*, *Il1b*, *Cxcl2*, and *Ccl7* and the catabolic enzymes *Mmp9* and *Mmp13* (Figure 4G). Box plots of the expression levels of the eight most highly upregulated genes are displayed in Supplementary Figure S8B. In contrast, 40 genes (80%) were not differentially expressed in limb tendon macrophages compared to tail tendon macrophages at P56. These data indicate that the phenotype of tendon resident macrophages is dependent on age and tendon, with limb tendon macrophages changing more than tail tendon macrophages with age.

### 3.5 Tendon resident macrophages and fibroblasts express compatible paracrine signaling genes

**FIGURE 5:**
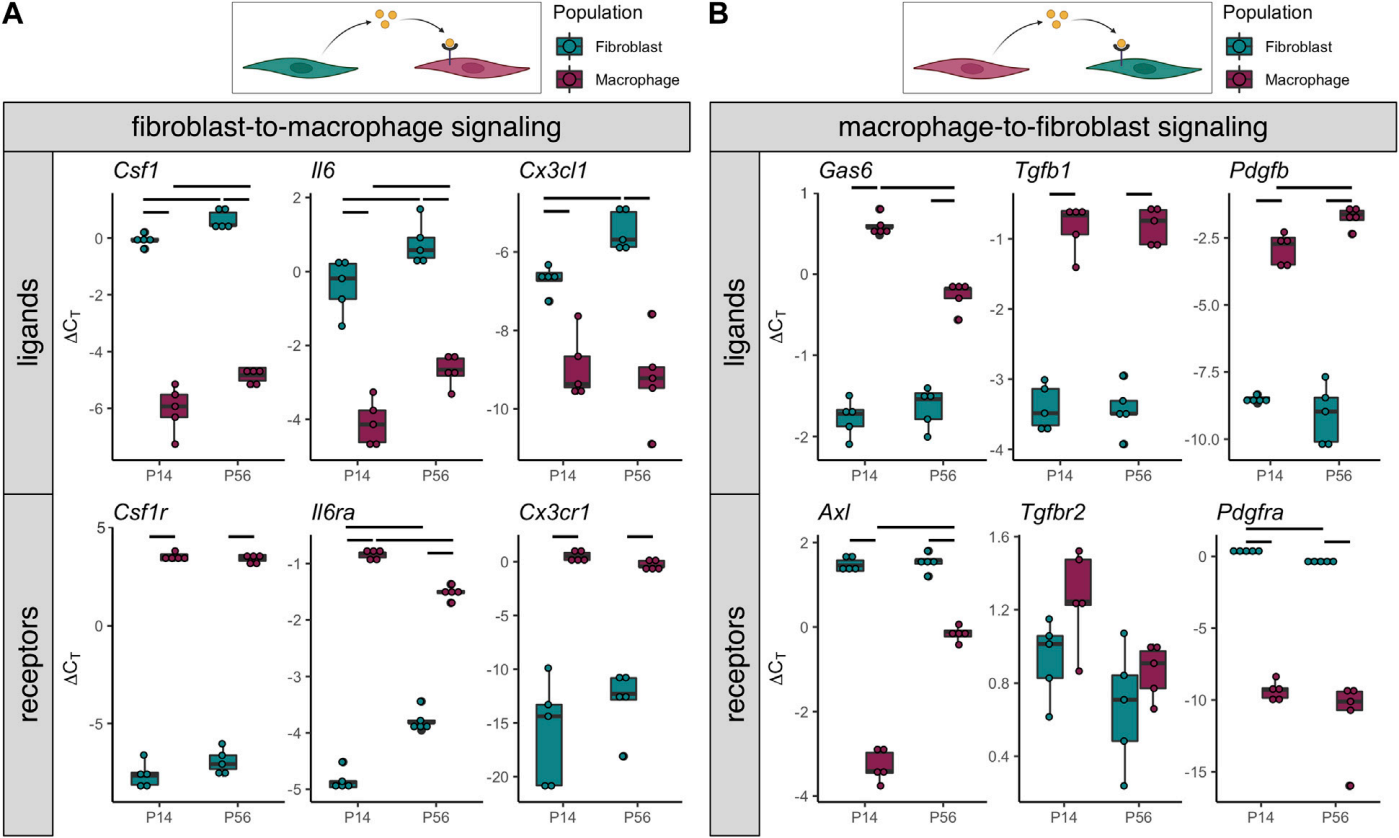
Gene expression indicates potential tendon resident macrophage-fibroblast crosstalk **(A)** ∆C_T_ levels of ligands enriched in tdTomato^+^ fibroblasts (top) and receptors enriched in F4/80-Brilliant Violet 421^+^ macrophages (bottom) from P14 and P56 limb tendons (*n* = 5) **(B)** ∆C_T_ levels of ligands enriched in F4/80-Brilliant Violet 421^+^ macrophages (top) and receptors enriched in tdTomato^+^ fibroblasts (bottom) from P14 and P56 limb tendons (*n* = 5). Significance bars indicate *p* < 0.05 as determined by Mann-Whitney U test.

Crosstalk between resident macrophages and the adjacent stromal cells (e.g., fibroblasts) within the tissue or organ plays key roles in development, growth, homeostasis, aging, and healing (Buechler et al., 2021; Franklin, 2021; Lee and Ginhoux, 2022). Based on these findings in other tissues and the intimate positioning of resident macrophages adjacent to tendon fibroblasts, we investigated the expression of various genes associated with paracrine signaling between F4/80^+^ macrophages and tdTomato^+^ fibroblasts. We found high expression of several ligands in fibroblasts with expression of corresponding receptors in macrophages (Figure 5A). As expected, one of the top potential fibroblast-to-macrophage ligand-receptor pairs was *Csf1-Csf1r*. CSF1R signaling is essential for the differentiation, survival, and function of most resident macrophage populations (Cecchini et al., 1994; Dai et al., 2002; Guilliams et al., 2020). Additionally, *Il6*-*Il6ra* was another potential fibroblast-to-macrophage ligand-receptor pairing. IL-6 is associated with pro-inflammatory signaling and is often induced by the NF-kB pathway. We also found evidence of *Cx3cl1*-*Cx3cr1* fibroblast-to-macrophage signaling. This suggests fibroblasts may drive chemotaxis of macrophages through CX3CL1 secretion.

Furthermore, we found that macrophages express several ligands whose associated receptors are expressed by fibroblasts (Figure 5B). The top potential macrophage-to-fibroblast ligand-receptor pairs were *Gas6*-*Axl*, *Tgfb1*-*Tgfbr2*, and *Pdgfb*-*Pdgfra*. GAS6-AXL signaling has been shown to regulate cell proliferation and survival, while TGF-B1 and PDGF-B are known growth factors with established roles in tendon fibroblast proliferation and differentiation. These data provide evidence that resident macrophages may regulate tendon fibroblast processes during postnatal growth through paracrine signaling.

### 3.6 Tendon resident macrophages preferentially localize to *Csf1*-expressing fibroblasts

In several tissues, disruption of local CSF1 expression by neighboring cells (e.g., fibroblasts) results in depletion of the resident macrophage population (Mondor et al., 2019; Bellomo et al., 2020; Werner et al., 2020; Emoto et al., 2022; Zhou et al., 2022). We re-analyzed two publicly available scRNA-seq datasets to investigate *Csf1* expression in the tendon cell population (Supplementary Figure S6) (Harvey et al., 2019; Tan et al., 2020). We found that *Csf1* was exclusively expressed in fibroblast clusters (Supplementary Figures S6E,H). Furthermore, we found that only a subset of fibroblasts expressed *Csf1*. In agreement with our findings, *Csf1r* was expressed in the macrophages and not in the fibroblasts (Supplementary Figures S6E,H).

**FIGURE 6:**
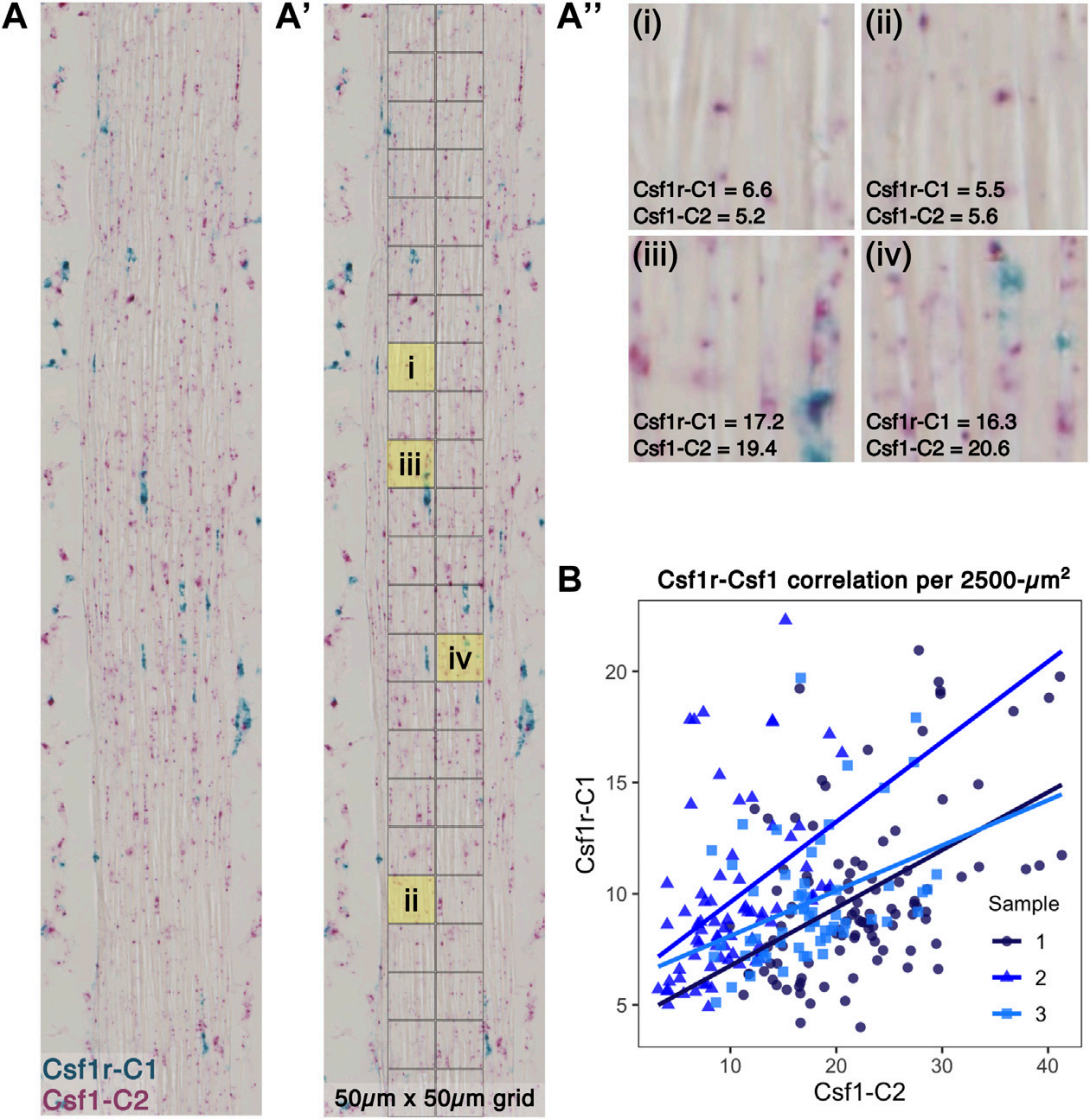
*Csf1r*-expressing macrophages localize to *Csf1*-expressing tendon fibroblasts **(A)** Representative image of RNAScope ISH of *Csf1* and *Csf1r* RNA on P28 patellar tendon sections. Scale bar = 20 µm **(A')** Each ISH image was divided into a 50 μm × 50 µm grid to quantify average staining intensity of each stain per unit area **(A'')** Representative 2,500-µm^2^ units with corresponding Csf1r-C1 and Csf1-C2 mean intensity measurements (low expression in upper row and high expression in lower row) **(B)** Scatter plot showing average intensity of Csf1r-C1 and Csf1-C2 staining within each unit area (*n* = 3).

To investigate the spatial relationship between *Csf1*-expressing fibroblasts and *Csf1r*-expressing macrophages within the tendon midsubstance, we performed RNAScope duplex *in situ* hybridization (ISH) on P28 sagittal patellar tendon cryosections (Figure 6A). We overlaid a 50 μm × 50 µm grid pattern over the tendon (Figure 6A') and then quantified the *Csf1r* and *Csf1* staining intensities within each area by performing color deconvolution to isolate individual channels for *Csf1r*, *Csf1*, and background signals (Figure 6A''; Supplementary Figure S10A). We found a significant positive correlation between *Csf1r* and *Csf1* staining (Figure 6B; mean Spearman’s rank correlation coefficient = 0.439, mean *p* < 0.001). There was no significant correlation between *Csf1* and Hoechst signals (mean Spearman’s rank correlation coefficient = −0.130, mean *p* = 0.361) or between *Csf1r* and Hoechst signals (mean Spearman’s rank correlation coefficient = −0.300, mean *p* = 0.574), suggesting that the *Csf1*-*Csf1r* correlation was not simply a function of cell density (Supplementary Figures S10B,C). Furthermore, we found low *Csf1* and *Csf1r* expression in the regions where we found few Csf1rGFP^+^ cells (Supplementary Figure S3): the patella-patellar tendon attachment (“pat”), tibia-patellar tendon attachment (“tib”), cruciate ligaments (“PCL”), and calcaneus-Achilles tendon attachment (“calc”) (Supplementary Figure S11). These results indicate that *Csf1* expression by tendon fibroblasts may dictate the positioning of *Csf1r*-expressing resident macrophages.

### 3.7 Tendon resident macrophages express genes associated with ECM interactions and internalize collagen

**FIGURE 7:**
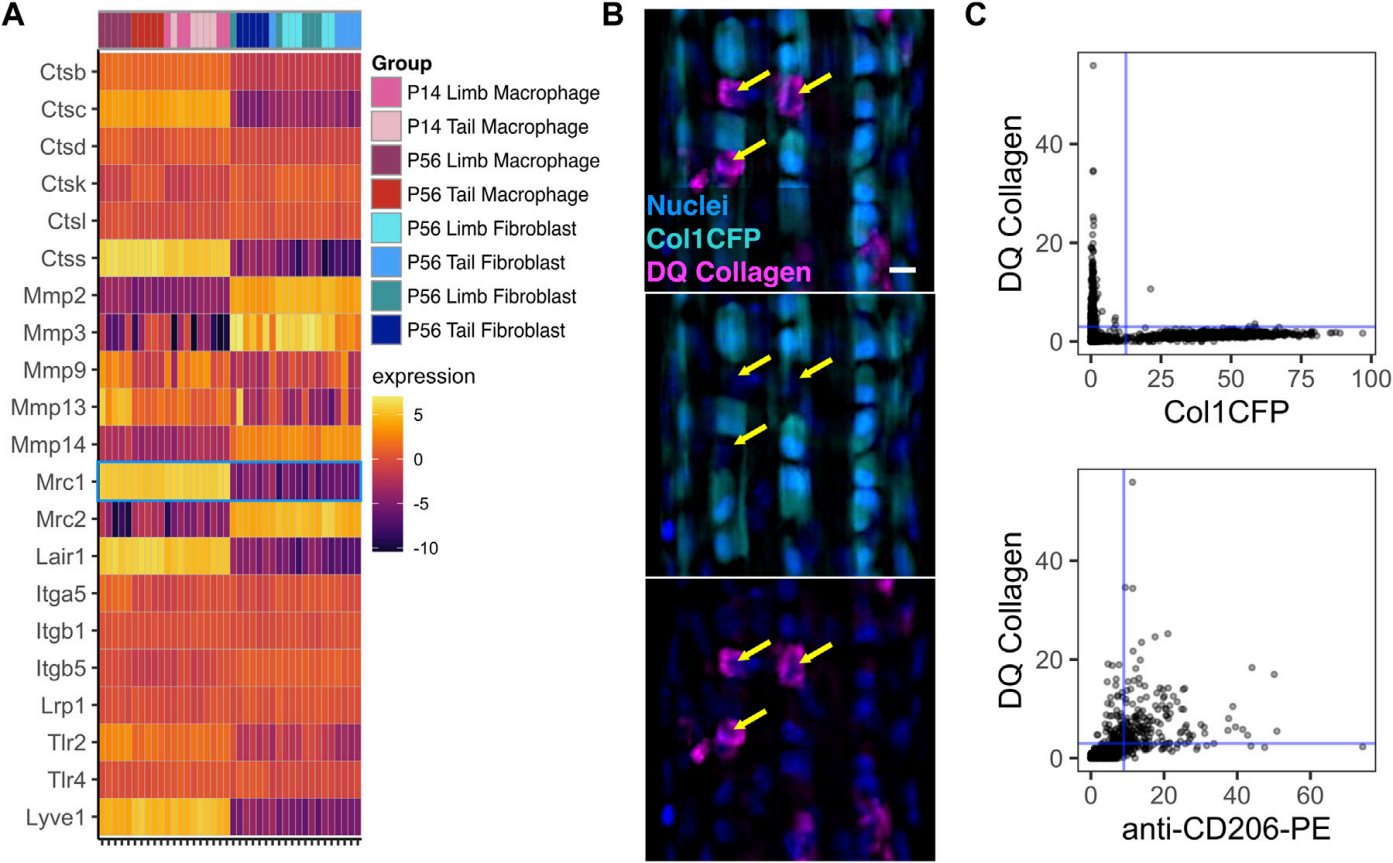
Tendon resident macrophages express ECM remodeling genes and internalize type I collagen **(A)** Heatmap of ECM-related genes. *Mrc1* (CD206) is outlined in blue **(B)** Representative image of Col1CFP tail tendons cultured with DQ Type I Collagen conjugated with fluorescein. Yellow arrows indicate Col1CFP^−^cells with internal DQ Collagen signal. Scale bar = 10 µm **(C)** Quantification of DQ Collagen vs Col1CFP signal intensity (top) and DQ Collagen vs. anti-CD206-PE signal intensity (bottom) in cells isolated from tail tendon explants.

Macrophages play key roles in directly remodeling the ECM during the development and growth of various tissues (Pollard, 2009; Wynn et al., 2013). Therefore, we investigated the expression of various genes associated with cell-ECM interactions (Figure 7A). Several cathepsins (*Ctsb*, *Ctsd*, *Ctsk*, and *Ctsl*) along with *Mmp9* were expressed at similar levels in both macrophages and fibroblasts. Furthermore, both macrophages and fibroblasts expressed integrin genes associated with cell-ECM adhesion and signaling (*Itga5*, *Itgb1*, and *Itgb5*) as well as receptors for the tendon ECM components decorin and biglycan (*Lrp1* and *Tlr4*, respectively). Several genes associated with collagen degradation (*Mmp2*, *Mmp3*, *Mmp14*, and *Mrc2*) were upregulated in fibroblasts compared to macrophages. Conversely, the catabolic genes *Ctsc*, *Ctss*, and *Mmp13* were selectively upregulated in macrophages. Receptors for the tendon ECM components collagen (*Lair1*), biglycan (*Tlr2*), and hyaluronan (*Lyve1*) were also upregulated in macrophages compared to fibroblasts.

*Mrc1*, which encodes the mannose receptor CD206, is highly expressed in tendon resident macrophages (Figure 7A; Figure 1G). In dermal macrophages, CD206 is essential for receptor-mediated endocytosis of collagen and subsequent lysosomal degradation (Madsen et al., 2013). To investigate the relationship between Col1CFP^−^ CD206^+^ macrophages and internalized collagen, we cultured Col1CFP tail tendon explants with medium containing fluorescein-conjugated DQ Collagen, which fluoresces upon degradation. We found strong intracellular DQ Collagen signal in the Col1CFP^−^ cells, but not in Col1CFP^+^ cells (Figure 7B). To quantify the colocalization of Col1CFP, CD206, and DQ collagen, we isolated single cells from the explants and immunostained them with anti-CD206-PE. As expected, CD206^+^ Col1CFP^+^ cells were extremely rare (0.4% of Col1CFP^+^ cells; Supplementary Figure S12A); F4/80^+^ Col1CFP^+^ cells were also rare (1.3% of Col1CFP^+^ cells; Supplementary Figure S12B). Of the CD206^+^ cells, 75.4% were positive for DQ collagen, compared to 0.5% of Col1CFP^+^ cells (Figure 7C). Additionally, 67.9% of F4/80^+^ cells were positive for DQ collagen (Supplementary Figure S12C). This indicates that tendon resident macrophages may internalize degraded collagen in a CD206-dependent manner. Altogether, these data provide insight into the potential functions of resident macrophages in tendon growth and homeostasis.

## 4 Discussion

This study established the spatiotemporal distribution and phenotype of tendon resident macrophages during development and growth. Tendon resident macrophages are present during tendon formation (E15.5) and increase in number dramatically during early postnatal growth, which coincides with tremendous ECM accrual. Macrophages are positioned adjacent to tendon fibroblasts within linear arrays in the tendon fascicle and gene expression analyses suggest signaling crosstalk between these populations. In fact, our duplex RNA *in situ* hybridization demonstrated that the positioning of macrophages within the tendon correlated with expression of *Csf1* by tendon fibroblasts. Lastly, resident macrophages within tendon explants internalize collagen to a greater degree than adjacent tendon fibroblasts, a process which may be mediated by the mannose receptor (CD206), and potentially a role for macrophages in ECM remodeling during tendon growth.

Resident macrophages play key roles in the development and homeostasis of various tissues and organs (Wynn et al., 2013; Wu and Hirschi, 2020; Lee and Ginhoux, 2022). While embryonic macrophage precursors share a common phenotype, this phenotype begins to diverge once the cells colonize their respective tissues of residence and differentiate into more specialized cells based on biological and physical signals from their local niche (Mass et al., 2016; Mass, 2018; T'Jonck et al., 2018). We found that resident macrophages position themselves adjacent to tendon fibroblasts as early as E15.5 in the patellar tendon (Figure 1B) and the proportion of resident macrophages increases over the course of early postnatal growth (Figure 1C; Figure 3B). This coincides with previous studies demonstrating a high rate of overall proliferation and an increase in ECM density and maturity over the same period (Ansorge et al., 2011; Grinstein et al., 2019). Based on our EdU proliferation analysis, macrophages within the tendon fascicle out-proliferate fibroblasts during early postnatal development (Figure 3), likely driving the rapid increase in the ratio of macrophages to fibroblasts. This is similar to Langerhans cells, Kupffer cells, alveolar macrophages, and microglia, where the vast majority of cells are long-lived or self-renewing resident macrophages derived from yolk sac erythromyeloid progenitors or fetal liver monocytes and not from circulating bone marrow-derived monocytes, as with kidney, cardiac, lung interstitial, and brain border-associated macrophages (Gomez Perdiguero et al., 2015; Dick et al., 2022). Furthermore, we confirmed that tendon resident macrophages express *Lyve1* and *Folr2*. Macrophages expressing these markers, termed “TLF^+^” macrophages, were found to be long-lived or self-renewing embryonically derived macrophages using genetic fate mapping and parabiosis studies (Dick et al., 2022); similar to tendon resident macrophages, TLF^+^ macrophages in the heart, liver, lung, kidney and brain are enriched for *Mrc1* (CD206). We also found that tendon resident macrophages express *Ccr2*, similar to a recent report using CCR2-GFP mice (Muscat et al., 2022), which is typically a marker of resident macrophages derived from circulating monocytes (Chakarov et al., 2019; Dick et al., 2022). Thus, genetic fate mapping and parabiosis studies are still needed to define the ontogeny of tendon resident macrophages throughout multiple stages of life. In other tissues, resident macrophages derived from circulating monocytes have different functions in homeostasis and response to injury compared to those derived from embryonic progenitors (Wang et al., 2020a; Weinberger et al., 2020). Future studies will investigate the presence of monocyte-derived macrophages in tendon and how their function differs from embryonically derived macrophages.

Via flow cytometry and gene expression analyses, we found that tendon resident macrophages express a number of pan-macrophage and resident macrophage markers (*Adgre1*, *CD86*, *Mrc1*, *Csf1r*, *Lyve1*, *Lyz2*, *Fcer1g*, *Pf4*, *Folr2*, *Cx3cr1*, and *Ccr2*) at multiple ages. Interestingly, we found that the expression profile of these cells changed with age, depending on the tendon. For instance, we found that, during the active growth phase (P14), resident macrophages from different tendons had similar gene expression profiles, providing evidence for a common early macrophage phenotype before the tendons reach maturity. Furthermore, the expression profile of adult limb tendon resident macrophages (P56) diverged from that of macrophages in younger mice, suggesting that they acquired a specialized state during growth and maturation, unlike tail tendon macrophages that displayed minimal expression changes with age in the set of genes analyzed. Our finding that macrophage gene expression in limb tendons differs from that in tail tendons is in agreement with the finding that transcriptomes differ across functionally different tendons (Disser et al., 2020). ECM properties and mechanical loading properties also differ across tendons (Birch, 2007; Choi et al., 2018). Therefore, it is conceivable that the crosstalk between fibroblasts and macrophages and their interactions with the ECM, unique to that specific tendon, dictates their phenotype.

Two models of spatial patterning may explain the distribution of tendon resident macrophages within the midsubstance: the contact inhibition model (“territory model”) or the “nurturing scaffold” model. In the contact inhibition model, such as has been proposed in *Drosophila* embryos, patterning is governed by mutual repulsion of neighboring macrophages (Stramer et al., 2010; Hume et al., 2019). In support of this model in tendon, while the proportion of resident macrophages increased with time, the number of macrophages per unit area remained relatively constant. Tendon resident macrophages are rarely found in close proximity to each other; instead, they are regularly patterned across the length and depth of the midsubstance. We found that tendon resident macrophages typically have cytoplasmic projections that reach around neighboring cells and collagen fibers. It is possible that, as the tendon grows in volume, contact inhibition is lost and macrophages proliferate to fill the missing “territories.” Alternatively, tendon resident macrophage density and distribution may be a function of secretion of CSF1 by tendon fibroblasts that acts to create a “nurturing scaffold” (Guilliams et al., 2020). In monolayer co-cultures of fibroblasts and macrophages, a stable cell circuit exists where the macrophage-to-fibroblast ratio reaches an equilibrium over time, regardless of initial seeding density or transient supplementation with exogenous CSF1 or PDGFB (Zhou et al., 2018). In macrophage monocultures, initial seeding densities do not affect proliferation rate, as one would expect in a contact inhibition-dependent model of patterning, but CSF1 concentration significantly increases the proliferation rate. *In vivo*, stromal cell production of CSF1 is necessary for resident macrophage growth and survival (Bonnardel et al., 2019; Mondor et al., 2019; Bellomo et al., 2020; Werner et al., 2020; Zhou et al., 2022). Global depletion of functional CSF1 results in a significant reduction in the number of tendon resident macrophages (Cecchini et al., 1994; Harris et al., 2012). Injection of exogenous CSF1 in the circulation does not result in replenishment of the tendon resident macrophage population, suggesting that local production of CSF1 by fibroblasts is required for the establishment and maintenance of the tendon resident macrophage population (Ryan et al., 2001). In tendon, we found that average *Csf1* expression by fibroblasts increases from P14 to P56 (Figure 5A), concomitantly with the increase in the ratio of macrophages to fibroblasts in the patellar tendon. This fact, in combination with our finding that *Csf1r*-expressing macrophages preferentially localize to *Csf1*-expressing fibroblasts (Figure 6), supports the “nurturing scaffold” model for tendon resident macrophage patterning. Future studies will investigate the functional importance of the CSF1-CSF1R signaling axis in tendon using conditional knockout mouse models.

Within the patellar and Achilles tendons examined, resident macrophages were not present in the enthesis. Furthermore, macrophages were rarely found in the cruciate ligaments. The factors driving macrophages to populate the tendon midsubstance and not the enthesis or cruciate ligaments are unknown, but may include differences in fibroblast phenotype, ECM, or mechanical loading. Interestingly, the density of resident macrophages within the Achilles tendon increased closer to the myotendinous junction. *In situ* hybridization of knee and ankle sections reveals a lack of *Csf1* expression in the cruciate ligaments and in the entheses of patellar and Achilles tendons which likely results in the low density of macrophages in these areas. However, the mechanisms that regulate the regional heterogeneity in *Csf1* expression in fibroblasts have yet to be determined.

We demonstrated in this study that fibroblasts and macrophages may communicate with each other through various paracrine signaling pathways. Previous studies also found evidence of fibroblast-immune cell interactions during normal physiology and tendinopathy (De Micheli et al., 2020; Akbar et al., 2021; Stauber et al., 2021). Crosstalk between resident macrophages and stromal cells contributes to the development, function, and maintenance of several tissues (Wynn et al., 2013; Buechler et al., 2021; Franklin, 2021). We found that, in tendon fibroblasts, several cytokines, including *Csf1*, *Il6*, and *Cx3cl1*, are upregulated in the adult tendon compared to the actively growing tendon. In addition, *in situ* hybridization (Figure 6) and scRNA-seq analysis (Supplementary Figure S6) demonstrated that only a subset of tendon fibroblasts expresses detectable levels of *Csf1*. It is unknown if this subset is a unique fibroblast population or a temporary state that all fibroblasts can enter when subjected to specific stimuli. In tendon resident macrophages, there are a few cytokines, such as *Gas6*, that are downregulated in the mature tendon, while there are other cytokines, such as *Pdgfb*, that are upregulated. These changes in cell-cell signaling may be representative of the overall differences in tendon phenotype at these two different developmental time points. Investigation of the roles of fibroblast-macrophage crosstalk in tendon development, homeostasis, and pathology will close a wide gap in knowledge in tendon biology.

Resident macrophages also directly remodel and interact with the ECM of several tissues, including bone, lung, and mammary gland, during development and homeostasis (Wiktor-Jedrzejczak et al., 1990; Pollard, 2009; Wynn et al., 2013; Wang et al., 2020b; Keerthivasan et al., 2021). The increase in the proportion of tendon resident macrophages correlates with the increase in ECM density, which may be indicative of the importance of macrophages in regulating the ECM. In tendon, resident macrophages are enriched for *Mrc1* (mannose receptor; CD206), a marker typically associated with “M2” anti-inflammatory macrophages. CD206^+^ dermal macrophages endocytose collagen through the mannose receptor and clear the internalized collagen through lysosomal degradation (Madsen et al., 2013). Similarly, we show that CD206^+^ tendon resident macrophages internalize type 1 collagen (Figure 7). This suggests a potential role for tendon resident macrophages in ECM turnover. A portion of the collagen matrix is turned over on a daily basis in the homeostatic tendon (Chang et al., 2020). While tendon fibroblasts across tissues are known to phagocytose and degrade collagen (Everts et al., 2003; Chang et al., 2020), it is possible that tendon resident macrophages play a complementary role in daily clearance of collagen through phagocytosis, receptor-mediated collagen endocytosis, or both (Madsen et al., 2013). We found that macrophages upregulate *Ctss* (cathepsin S), which can degrade elastin (Vidak et al., 2019; Brown et al., 2020), and *Lair1* (leukocyte-associated immunoglobulin-like receptor one; CD305), which recognizes type 1 collagen (Carvalheiro et al., 2020; Keerthivasan et al., 2021). Additionally, we demonstrated that tendon resident macrophages express *Apoe* and *Lrp1*, which are both involved in type 1 collagen phagocytosis in alveolar macrophages (Cui et al., 2020). Lastly, tendon resident macrophages highly express *Lyve1*, a hyaluronan receptor. LYVE1^+^ resident macrophages in other tissues have been implicated in maintaining ECM homeostasis (Lim et al., 2018; Brezovakova and Jadhav, 2020; Wang et al., 2020b). Given the presence of ECM-related genes that are differentially expressed between tendon resident macrophages and tendon fibroblasts, a potential feedback loop may exist involving macrophages, fibroblasts, and the ECM to regulate normal tendon physiology. An understanding of how the dysregulation of the ECM, as seen in tendinopathy and tendon healing, affects the macrophage phenotype may lead to improved clinical treatments (Jürgensen et al., 2020; Crosio and Huang, 2022). One recent study utilizing single-cell RNA sequencing found major compositional and transcriptional differences in the immune cell population between healthy and tendinopathic human tendons (Akbar et al., 2021). There is therefore a critical need to study how the resident macrophage population may contribute to healing following acute injury and to chronic tendinopathy.

Our results provide vital insights into the resident macrophage population during tendon development and growth. We have presented evidence of crosstalk between resident macrophages and fibroblasts and between resident macrophages and the surrounding matrix. Future studies will further investigate the dependence of tendon fibroblasts on signals from neighboring resident macrophages and the interactions of resident macrophages with the ECM during tendon growth, homeostasis, and tendinopathy.

## Data Availability

The datasets presented in this study can be found in online repositories. The names of the repository/repositories and accession number(s) can be found in the article/Supplementary Material.

## References

[B1] Akbar M. Macdonald L. Crowe L. A. N. Carlberg K. Kurowska-Stolarska M. Stahl P. L. (2021). Single cell and spatial transcriptomics in human tendon disease indicate dysregulated immune homeostasis. Ann. Rheum. Dis. 80, 1494–1497. 10.1136/annrheumdis-2021-220256 34001518 PMC8522454

[B2] Ansorge H. L. Adams S. Birk D. E. Soslowsky L. J. (2011). Mechanical, compositional, and structural properties of the post-natal mouse Achilles tendon. Ann. Biomed. Eng. 39, 1904–1913. 10.1007/s10439-011-0299-0 21431455 PMC3341172

[B3] Bellomo A. Mondor I. Spinelli L. Lagueyrie M. Stewart B. J. Brouilly N. (2020). Reticular fibroblasts expressing the transcription factor WT1 define a stromal niche that maintains and replenishes splenic red pulp macrophages. Immunity 53, 127–142. 10.1016/j.immuni.2020.06.008 32562599

[B4] Best K. T. Loiselle A. E. (2019). Scleraxis lineage cells contribute to organized bridging tissue during tendon healing and identify a subpopulation of resident tendon cells. FASEB J. 33, 8578–8587. 10.1096/fj.201900130RR 30951381 PMC6593880

[B5] Birch H. L. (2007). Tendon matrix composition and turnover in relation to functional requirements. Int. J. Exp. Pathol. 88, 241–248. 10.1111/j.1365-2613.2007.00552.x 17696905 PMC2517317

[B6] Bleriot C. Chakarov S. Ginhoux F. (2020). Determinants of resident tissue macrophage identity and function. Immunity 52, 957–970. 10.1016/j.immuni.2020.05.014 32553181

[B7] Blitz E. Sharir A. Akiyama H. Zelzer E. (2013). Tendon-bone attachment unit is formed modularly by a distinct pool of Scx- and Sox9-positive progenitors. Development 140, 2680–2690. 10.1242/dev.093906 23720048

[B8] Bonnardel J. T'Jonck W. Gaublomme D. Browaeys R. Scott C. L. Martens L. (2019). Stellate cells, hepatocytes, and endothelial cells imprint the kupffer cell identity on monocytes colonizing the liver macrophage niche. Immunity 51, 638–654. 10.1016/j.immuni.2019.08.017 31561945 PMC6876284

[B9] Brezovakova V. Jadhav S. (2020). Identification of Lyve-1 positive macrophages as resident cells in meninges of rats. J. Comp. Neurol. 528, 2021–2032. 10.1002/cne.24870 32003471

[B10] Brown R. Nath S. Lora A. Samaha G. Elgamal Z. Kaiser R. (2020). Cathepsin S: Investigating an old player in lung disease pathogenesis, comorbidities, and potential therapeutics. Respir. Res. 21, 111. 10.1186/s12931-020-01381-5 32398133 PMC7216426

[B11] Buechler M. B. Fu W. Turley S. J. (2021). Fibroblast-macrophage reciprocal interactions in health, fibrosis, and cancer. Immunity 54, 903–915. 10.1016/j.immuni.2021.04.021 33979587

[B12] Carvalheiro T. Garcia S. Pascoal Ramos M. I. Giovannone B. Radstake T. Marut W. (2020). Leukocyte associated immunoglobulin like receptor 1 regulation and function on monocytes and dendritic cells during inflammation. Front. Immunol. 11, 1793. 10.3389/fimmu.2020.01793 32973751 PMC7466540

[B13] Cecchini M. G. Dominguez M. G. Mocci S. Wetterwald A. Felix R. Fleisch H. (1994). Role of colony stimulating factor-1 in the establishment and regulation of tissue macrophages during postnatal development of the mouse. Development 120, 1357–1372. 10.1242/dev.120.6.1357 8050349

[B14] Chakarov S. Lim H. Y. Tan L. Lim S. Y. See P. Lum J. (2019). Two distinct interstitial macrophage populations coexist across tissues in specific subtissular niches. Science 363, eaau0964. 10.1126/science.aau0964 30872492

[B15] Chang J. Garva R. Pickard A. Yeung C. C. Mallikarjun V. Swift J. (2020). Circadian control of the secretory pathway maintains collagen homeostasis. Nat. Cell Biol. 22, 74–86. 10.1038/s41556-019-0441-z 31907414 PMC7613259

[B16] Choi H. Simpson D. Wang D. Prescott M. Pitsillides A. A. Dudhia J. (2020). Heterogeneity of proteome dynamics between connective tissue phases of adult tendon. Elife 9, e55262. 10.7554/eLife.55262 32393437 PMC7217697

[B17] Choi R. Smith M. Clarke E. Little C. (2018). Cellular, matrix, and mechano-biological differences in load-bearing versus positional tendons throughout development and aging: A narrative review. Connect. Tissue Res. 59, 483–494. 10.1080/03008207.2018.1504929 30231648

[B18] Crosio G. Huang A. H. (2022). Innate and adaptive immune system cells implicated in tendon healing and disease. Eur. Cells Mater. 43, 39–52. 10.22203/eCM.v043a05 PMC9526522 35178698

[B19] Cui H. Jiang D. Banerjee S. Xie N. Kulkarni T. Liu R. M. (2020). Monocyte-derived alveolar macrophage apolipoprotein E participates in pulmonary fibrosis resolution. JCI Insight 5, e134539. 10.1172/jci.insight.134539 32027623 PMC7141408

[B20] Dai X. M. Ryan G. R. Hapel A. J. Dominguez M. G. Russell R. G. Kapp S. (2002). Targeted disruption of the mouse colony-stimulating factor 1 receptor gene results in osteopetrosis, mononuclear phagocyte deficiency, increased primitive progenitor cell frequencies, and reproductive defects. Blood 99, 111–120. 10.1182/blood.v99.1.111 11756160

[B21] De Micheli A. J. Swanson J. B. Disser N. P. Martinez L. M. Walker N. R. Oliver D. J. (2020). Single-cell transcriptomic analysis identifies extensive heterogeneity in the cellular composition of mouse Achilles tendons. Am. J. Physiol. Cell Physiol. 319, C885–C894. 10.1152/ajpcell.00372.2020 32877217 PMC7701267

[B22] Dick S. A. Wong A. Hamidzada H. Nejat S. Nechanitzky R. Vohra S. (2022). Three tissue resident macrophage subsets coexist across organs with conserved origins and life cycles. Sci. Immunol. 7, eabf7777. 10.1126/sciimmunol.abf7777 34995099

[B23] Disser N. P. Ghahramani G. C. Swanson J. B. Wada S. Chao M. L. Rodeo S. A. (2020). Widespread diversity in the transcriptomes of functionally divergent limb tendons. J. Physiol. 598, 1537–1550. 10.1113/JP279646 32083717 PMC7351251

[B24] Dyment N. A. Jiang X. Chen L. Hong S. H. Adams D. J. Ackert-Bicknell C. (2016). High-Throughput, multi-image cryohistology of mineralized tissues. J. Vis. Exp. 14, 54468. 10.3791/54468 PMC5092021 27684089

[B25] Emoto T. Lu J. Sivasubramaniyam T. Maan H. Khan A. B. Abow A. A. (2022). Colony stimulating factor-1 producing endothelial cells and mesenchymal stromal cells maintain monocytes within a perivascular bone marrow niche. Immunity 55, 862–878. e8. 10.1016/j.immuni.2022.04.005 35508166

[B26] Everts V. Hou W. S. Rialland X. Tigchelaar W. Saftig P. Bromme D. (2003). Cathepsin K deficiency in pycnodysostosis results in accumulation of non-digested phagocytosed collagen in fibroblasts. Calcif. Tissue Int. 73, 380–386. 10.1007/s00223-002-2092-4 12874701

[B27] Franklin R. A. (2021). Fibroblasts and macrophages: Collaborators in tissue homeostasis. Immunol. Rev. 302, 86–103. 10.1111/imr.12989 34101202

[B28] Gomez Perdiguero E. Klapproth K. Schulz C. Busch K. Azzoni E. Crozet L. (2015). Tissue-resident macrophages originate from yolk-sac-derived erythro-myeloid progenitors. Nature 518, 547–551. 10.1038/nature13989 25470051 PMC5997177

[B29] Grinstein M. Dingwall H. L. O'Connor L. D. Zou K. Capellini T. D. Galloway J. L. (2019). A distinct transition from cell growth to physiological homeostasis in the tendon. Elife 8, e48689. 10.7554/eLife.48689 31535975 PMC6791717

[B30] Guilliams M. Thierry G. R. Bonnardel J. Bajenoff M. (2020). Establishment and maintenance of the macrophage niche. Immunity 52, 434–451. 10.1016/j.immuni.2020.02.015 32187515

[B31] Harris S. E. Macdougall M. Horn D. Woodruff K. Zimmer S. N. Rebel V. I. (2012). Meox2Cre-mediated disruption of CSF-1 leads to osteopetrosis and osteocyte defects. Bone 50, 42–53. 10.1016/j.bone.2011.09.038 21958845 PMC3374485

[B32] Harvey T. Flamenco S. Fan C. M. (2019). A Tppp3(+)Pdgfra(+) tendon stem cell population contributes to regeneration and reveals a shared role for PDGF signalling in regeneration and fibrosis. Nat. Cell Biol. 21, 1490–1503. 10.1038/s41556-019-0417-z 31768046 PMC6895435

[B33] Heinemeier K. M. Schjerling P. Heinemeier J. Magnusson S. P. Kjaer M. (2013). Lack of tissue renewal in human adult Achilles tendon is revealed by nuclear bomb (14)C. FASEB J. 27, 2074–2079. 10.1096/fj.12-225599 23401563 PMC3633810

[B34] Hoeffel G. Chen J. Lavin Y. Low D. Almeida F. F. See P. (2015). C-Myb(+) erythro-myeloid progenitor-derived fetal monocytes give rise to adult tissue-resident macrophages. Immunity 42, 665–678. 10.1016/j.immuni.2015.03.011 25902481 PMC4545768

[B35] Hume D. A. Irvine K. M. Pridans C. (2019). The mononuclear phagocyte system: The relationship between monocytes and macrophages. Trends Immunol. 40, 98–112. 10.1016/j.it.2018.11.007 30579704

[B36] Ingman W. V. Wyckoff J. Gouon-Evans V. Condeelis J. Pollard J. W. (2006). Macrophages promote collagen fibrillogenesis around terminal end buds of the developing mammary gland. Dev. Dyn. 235, 3222–3229. 10.1002/dvdy.20972 17029292

[B37] Jürgensen H. J. Van Putten S. Nørregaard K. S. Bugge T. H. Engelholm L. H. Behrendt N. (2020). Cellular uptake of collagens and implications for immune cell regulation in disease. Cell. Mol. Life Sci. 77, 3161–3176. 10.1007/s00018-020-03481-3 32100084 PMC11105017

[B38] Kalajzic I. Kalajzic Z. Kaliterna M. Gronowicz G. Clark S. H. Lichtler A. C. (2002). Use of type I collagen green fluorescent protein transgenes to identify subpopulations of cells at different stages of the osteoblast lineage. J. Bone Min. Res. 17, 15–25. 10.1359/jbmr.2002.17.1.15 11771662

[B39] Keerthivasan S. Şenbabaoğlu Y. Martinez-Martin N. Husain B. Verschueren E. Wong A. (2021). Homeostatic functions of monocytes and interstitial lung macrophages are regulated via collagen domain-binding receptor LAIR1. Immunity 54, 1511–1526. e8. 10.1016/j.immuni.2021.06.012 34260887

[B40] Koyama E. Mundy C. Saunders C. Chung J. Catheline S. E. Rux D. (2021). Premature growth plate closure caused by a hedgehog cancer drug is preventable by Co-administration of a retinoid antagonist in mice. J. Bone Min. Res. 36, 1387–1402. 10.1002/jbmr.4291 PMC9661967 33724538

[B41] Lee C. Z. W. Ginhoux F. (2022). Biology of resident tissue macrophages. Development 149. 10.1242/dev.200270 35502781

[B42] Lehner C. Spitzer G. Gehwolf R. Wagner A. Weissenbacher N. Deininger C. (2019). Tenophages: A novel macrophage-like tendon cell population expressing CX3CL1 and CX3CR1. Dis. Model Mech. 12, dmm041384. 10.1242/dmm.041384 31744815 PMC6918766

[B43] Lim H. Y. Lim S. Y. Tan C. K. Thiam C. H. Goh C. C. Carbajo D. (2018). Hyaluronan receptor LYVE-1-expressing macrophages maintain arterial tone through hyaluronan-mediated regulation of smooth muscle cell collagen. Immunity 49, 326–341. 10.1016/j.immuni.2018.06.008 30054204

[B44] Liu C. F. Aschbacher-Smith L. Barthelery N. J. Dyment N. Butler D. Wylie C. (2012). Spatial and temporal expression of molecular markers and cell signals during normal development of the mouse patellar tendon. Tissue Eng. Part A 18, 598–608. 10.1089/ten.TEA.2011.0338 21939397 PMC3286855

[B45] Madisen L. Zwingman T. A. Sunkin S. M. Oh S. W. Zariwala H. A. Gu H. (2010). A robust and high-throughput Cre reporting and characterization system for the whole mouse brain. Nat. Neurosci. 13, 133–140. 10.1038/nn.2467 20023653 PMC2840225

[B46] Madsen D. H. Leonard D. Masedunskas A. Moyer A. Jürgensen H. J. Peters D. E. (2013). M2-like macrophages are responsible for collagen degradation through a mannose receptor-mediated pathway. J. Cell Biol. 202, 951–966. 10.1083/jcb.201301081 24019537 PMC3776354

[B47] Mass E. (2018). Delineating the origins, developmental programs and homeostatic functions of tissue-resident macrophages. Int. Immunol. 30, 493–501. 10.1093/intimm/dxy044 29986024

[B48] Mass E. Ballesteros I. Farlik M. Halbritter F. Gunther P. Crozet L. (2016). Specification of tissue-resident macrophages during organogenesis. Science 353, aaf4238. 10.1126/science.aaf4238 27492475 PMC5066309

[B49] Metsalu T. Vilo J. (2015). ClustVis: A web tool for visualizing clustering of multivariate data using principal component analysis and heatmap. Nucleic Acids Res. 43, W566–W570. 10.1093/nar/gkv468 25969447 PMC4489295

[B50] Mondor I. Baratin M. Lagueyrie M. Saro L. Henri S. Gentek R. (2019). Lymphatic endothelial cells are essential components of the subcapsular sinus macrophage niche. Immunity 50, 1453–1466. 10.1016/j.immuni.2019.04.002 31053503 PMC6697131

[B51] Muscat S. Nichols A. E. C. Gira E. Loiselle A. E. (2022). CCR2 is expressed by tendon resident macrophage and T cells, while CCR2 deficiency impairs tendon healing via blunted involvement of tendon-resident and circulating monocytes/macrophages. Faseb J. 36, e22607. 10.1096/fj.202201162R 36250393 PMC9593314

[B52] Peck B. D. Murach K. A. Walton R. G. Simmons A. J. Long D. E. Kosmac K. (2022). A muscle cell-macrophage axis involving matrix metalloproteinase 14 facilitates extracellular matrix remodeling with mechanical loading. FASEB J. 36, e22155. 10.1096/fj.202100182RR 35044708 PMC8875325

[B53] Pollard J. W. (2009). Trophic macrophages in development and disease. Nat. Rev. Immunol. 9, 259–270. 10.1038/nri2528 19282852 PMC3648866

[B54] Pryce B. A. Brent A. E. Murchison N. D. Tabin C. J. Schweitzer R. (2007). Generation of transgenic tendon reporters, ScxGFP and ScxAP, using regulatory elements of the scleraxis gene. Dev. Dyn. 236, 1677–1682. 10.1002/dvdy.21179 17497702

[B55] Ryan G. R. Dai X. M. Dominguez M. G. Tong W. Chuan F. Chisholm O. (2001). Rescue of the colony-stimulating factor 1 (CSF-1)-nullizygous mouse (Csf1(op)/Csf1(op)) phenotype with a CSF-1 transgene and identification of sites of local CSF-1 synthesis. Blood 98, 74–84. 10.1182/blood.v98.1.74 11418465

[B56] Sasmono R. T. Oceandy D. Pollard J. W. Tong W. Pavli P. Wainwright B. J. (2003). A macrophage colony-stimulating factor receptor-green fluorescent protein transgene is expressed throughout the mononuclear phagocyte system of the mouse. Blood 101 (3), 1155–1163. 10.1182/blood-2002-02-0569 12393599

[B57] Sorkin M. Huber A. K. Hwang C. Carson W. F. T. Menon R. Li J. (2020). Regulation of heterotopic ossification by monocytes in a mouse model of aberrant wound healing. Nat. Commun. 11, 722. 10.1038/s41467-019-14172-4 32024825 PMC7002453

[B58] Stauber T. Wolleb M. Duss A. Jaeger P. K. Heggli I. Hussien A. A. (2021). Extrinsic macrophages protect while tendon progenitors degrade: Insights from a tissue engineered model of tendon compartmental crosstalk. Adv. Healthc. Mater 10, e2100741. 10.1002/adhm.202100741 34494401 PMC11468160

[B59] Stramer B. Moreira S. Millard T. Evans I. Huang C. Y. Sabet O. (2010). Clasp-mediated microtubule bundling regulates persistent motility and contact repulsion in Drosophila macrophages *in vivo* . J. Cell Biol. 189, 681–689. 10.1083/jcb.200912134 20457764 PMC2872918

[B60] Stuart T. Butler A. Hoffman P. Hafemeister C. Papalexi E. Mauck W. M. (2019). Comprehensive integration of single-cell data. Cell 177, 1888–1902. 10.1016/j.cell.2019.05.031 31178118 PMC6687398

[B61] T'Jonck W. Guilliams M. Bonnardel J. (2018). Niche signals and transcription factors involved in tissue-resident macrophage development. Cell. Immunol. 330, 43–53. 10.1016/j.cellimm.2018.02.005 29463401 PMC6108424

[B62] Tan G. K. Pryce B. A. Stabio A. Brigande J. V. Wang C. Xia Z. (2020). Tgfβ signaling is critical for maintenance of the tendon cell fate. Elife 9, e52695. 10.7554/eLife.52695 31961320 PMC7025861

[B63] Theret M. Mounier R. Rossi F. (2019). The origins and non-canonical functions of macrophages in development and regeneration. Dev. Camb. 146, dev156000–14. 10.1242/dev.156000 31048317

[B64] Thorpe C. T. Streeter I. Pinchbeck G. L. Goodship A. E. Clegg P. D. Birch H. L. (2010). Aspartic acid racemization and collagen degradation markers reveal an accumulation of damage in tendon collagen that is enhanced with aging. J. Biol. Chem. 285, 15674–15681. 10.1074/jbc.M109.077503 20308077 PMC2871433

[B65] Tsinman T. K. Jiang X. Han L. Koyama E. Mauck R. L. Dyment N. A. (2021). Intrinsic and growth-mediated cell and matrix specialization during murine meniscus tissue assembly. FASEB J. 35, e21779. 10.1096/fj.202100499R 34314047 PMC8323983

[B66] Vidak E. Javorsek U. Vizovisek M. Turk B. (2019). Cysteine cathepsins and their extracellular roles: Shaping the microenvironment. Cells 8, 264. 10.3390/cells8030264 30897858 PMC6468544

[B67] Wang X. Sathe A. A. Smith G. R. Ruf-Zamojski F. Nair V. Lavine K. J. (2020a). Heterogeneous origins and functions of mouse skeletal muscle-resident macrophages. Proc. Natl. Acad. Sci. U. S. A. 117 (34), 20729–20740. 10.1073/pnas.1915950117 32796104 PMC7456122

[B68] Wang Y. Chaffee T. S. Larue R. S. Huggins D. N. Witschen P. M. Ibrahim A. M. (2020b). Tissue-resident macrophages promote extracellular matrix homeostasis in the mammary gland stroma of nulliparous mice. Elife 9, e57438. 10.7554/eLife.57438 32479261 PMC7297528

[B69] Weinberger T. Esfandyari D. Messerer D. Percin G. Schleifer C. Thaler R. (2020). Ontogeny of arterial macrophages defines their functions in homeostasis and inflammation. Nat. Commun. 11, 4549. 10.1038/s41467-020-18287-x 32917889 PMC7486394

[B70] Werner S. L. Sharma R. Woodruff K. Horn D. Harris S. E. Gorin Y. (2020). CSF-1 in osteocytes inhibits nox4-mediated oxidative stress and promotes normal bone homeostasis. JBMR Plus 4, e10080. 10.1002/jbm4.10080 32666016 PMC7340444

[B71] Wiktor-Jedrzejczak W. Bartocci A. Ferrante A. W. JR. Ahmed-Ansari A. Sell K. W. Pollard J. W. (1990). Total absence of colony-stimulating factor 1 in the macrophage-deficient osteopetrotic (op/op) mouse. Proc. Natl. Acad. Sci. U. S. A. 87, 4828–4832. 10.1073/pnas.87.12.4828 2191302 PMC54211

[B72] Wu Y. Hirschi K. K. (2020). Tissue-resident macrophage development and function. Front. Cell Dev. Biol. 8, 617879. 10.3389/fcell.2020.617879 33490082 PMC7820365

[B73] Wynn T. A. Chawla A. Pollard J. W. (2013). Macrophage biology in development, homeostasis and disease. Nature 496, 445–455. 10.1038/nature12034 23619691 PMC3725458

[B74] Zhang C. Couppe C. Scheijen J. Schalkwijk C. G. Kjaer M. Magnusson S. P. (2020). Regional collagen turnover and composition of the human patellar tendon. J. Appl. Physiol. 128, 884–891. 10.1152/japplphysiol.00030.2020 32163333

[B75] Zhou X. Franklin R. A. Adler M. Carter T. S. Condiff E. Adams T. S. (2022). Microenvironmental sensing by fibroblasts controls macrophage population size. Proc. Natl. Acad. Sci. U. S. A. 119, e2205360119. 10.1073/pnas.2205360119 35930670 PMC9371703

[B76] Zhou X. Franklin R. A. Adler M. Jacox J. B. Bailis W. Shyer J. A. (2018). Circuit design features of a stable two-cell system. Cell 172, 744–757. 10.1016/j.cell.2018.01.015 29398113 PMC7377352

