# Supplementary material for: CD206+ tendon resident macrophages and their potential crosstalk with fibroblasts and the ECM during tendon growth and maturation
Source: Front Physiol. 2023 Feb 22;14:1122348. doi: 10.3389/fphys.2023.1122348 (PMC9992419; doi:10.3389/fphys.2023.1122348)
Supplement: Supplementary file 1 [file Presentation1.PDF]

Supplementary Material

1 Supplementary Tables

Table 1. List of TaqMan Gene Expression Assays used for Fluidigm high-throughput qPCR.

Gene	Assay ID
18s	Mm03928990_g1
Abl1	Mm00802029_m1
Adgre1	Mm00802529_m1
Apoe	Mm01307193_g1
Axl	Mm00437221_m1
Bgn	Mm01191753_m1
C1qa	Mm07295529_m1
C1qb	Mm01179619_m1
C1qc	Mm00776126_m1
Ccl2	Mm00441242_m1
Ccl7	Mm00443113_m1
Ccr2	Mm00438270_m1
Cd47	Mm00495011_m1
Cd68	Mm03047343_m1
Cd86	Mm00444540_m1
Cdh11	Mm00515466_m1
Cdh2	Mm01162497_m1
Clec10a	Mm00546125_g1
Col1a1	Mm00801666_g1
Col3a1	Mm01254476_m1
Col6a1	Mm00487160_m1
Col6a5	Mm01231908_m1
Colec12	Mm01236242_m1
Csf1	Mm00432686_m1
Csf1r	Mm01266652_m1
Csf2	Mm01290062_m1
Csf2ra	Mm00438331_g1
Ctsb	Mm01310506_m1
Ctsc	Mm00515580_m1
Ctsd	Mm00515586_m1
Ctsk	Mm00484039_m1
Ctsl	Mm00515597_m1
Ctss	Mm01255859_m1
Cx3cl1	Mm00436454_m1
Cx3cr1	Mm00438354_m1
Cxcl2	Mm00436450_m1
Dcn	Mm00514535_m1
Efnb2	Mm00438670_m1
Epha4	Mm01256007_m1
F13a1	Mm00472334_m1
Fcer1g	Mm02343757_m1
Folr2	Mm00433357_m1
Gas6	Mm00490378_m1
Gja1	Mm01179639_s1
Ifi27l2a	Mm07294121_m1
Igf1	Mm00439560_m1
Igf1r	Mm00802831_m1
Il10	Mm01288386_m1
Il10ra	Mm00434151_m1
Il1b	Mm00434228_m1
Il1r1	Mm00434237_m1
Il2	Mm00434256_m1
Il2ra	Mm01340213_m1
Il34	Mm01243248_m1
Il6	Mm00446190_m1
Il6ra	Mm01211445_m1
Itga5	Mm00439797_m1
Itgam	Mm00434455_m1
Itgb1	Mm01253230_m1
Itgb5	Mm00439825_m1
Lair1	Mm00618113_m1
Lrp1	Mm00464608_m1
Lyve1	Mm00475056_m1
Lyz2	Mm01612741_m1
Mki67	Mm01278617_m1

Mkx	Mm00617017_m1
Mmp13	Mm00439491_m1
Mmp14	Mm00485054_m1
Mmp2	Mm00439498_m1
Mmp3	Mm00440295_m1
Mmp9	Mm00442991_m1
Mrc1	Mm01329359_m1
Mrc2	Mm00485184_m1
Pcna	Mm00448100_g1
Pdgfb	Mm00440677_m1
Pdgfc	Mm00480205_m1
Pdgfra	Mm00440701_m1
Pf4	Mm00451315_g1
Plxnb2	Mm00507118_m1
Prg4	Mm01284582_m1
Rps17	Mm01314921_g1

Scx	Mm01205675_m1
Sema4c	Mm01229764_m1
Sirpa	Mm00455928_m1
Tgfb1	Mm01178820_m1
Tgfb2	Mm00436955_m1
Tgfb3	Mm00436960_m1
Tgfbr2	Mm03024091_m1
Tlr2	Mm00442346_m1
Tlr4	Mm00445273_m1
Tnf	Mm00443258_m1
Tnfrsf1b	Mm00441889_m1
Tnmd	Mm00491594_m1
Wls	Mm00509695_m1
Wnt5a	Mm00437347_m1
Wnt9a	Mm00460518_m1

2 Supplementary Figures

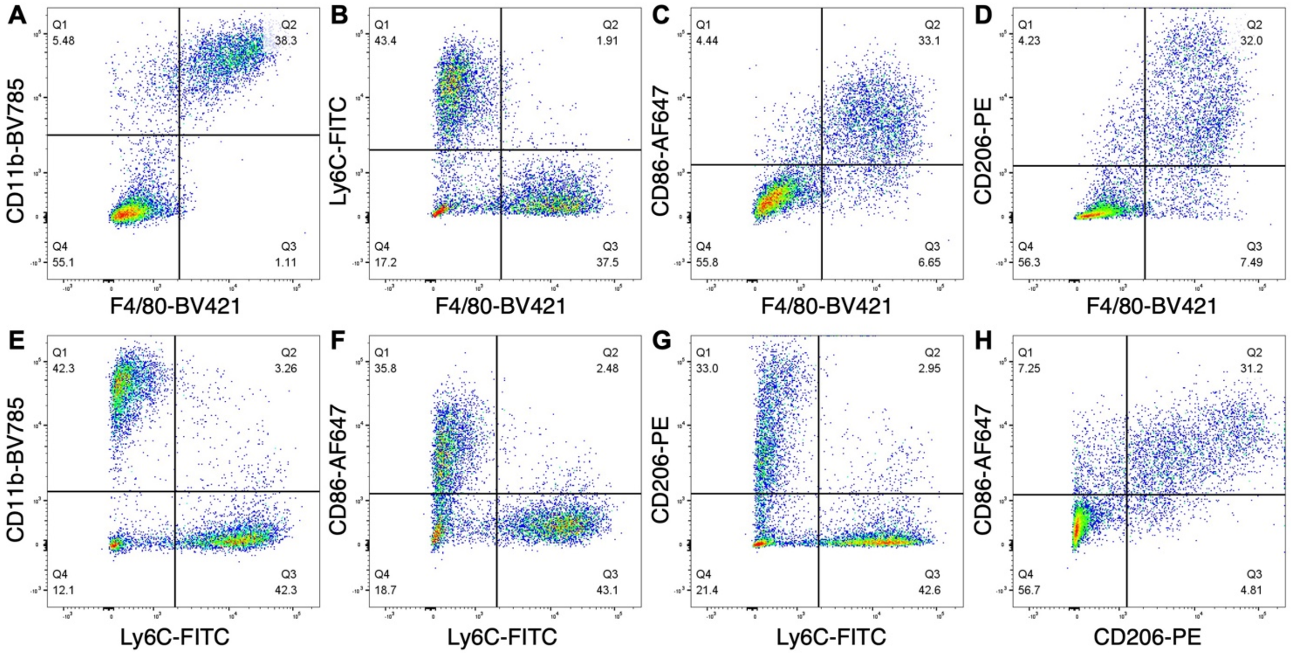

Supplementary Figure 1. Representative scatter plots of flow cytometry panel demonstrating marker expression within total tendon cell population. **(A-D)** Nearly all F4/80+ cells have similar expression levels of CD11b, Ly-6C, CD86, and CD206. CD206-PE staining had the greatest spread among F4/80+ cells. **(E-G)** Most Ly-6C+ cells were negative for CD11b, CD86, and CD206. **(H)** Most CD206+ cells were also CD86+.

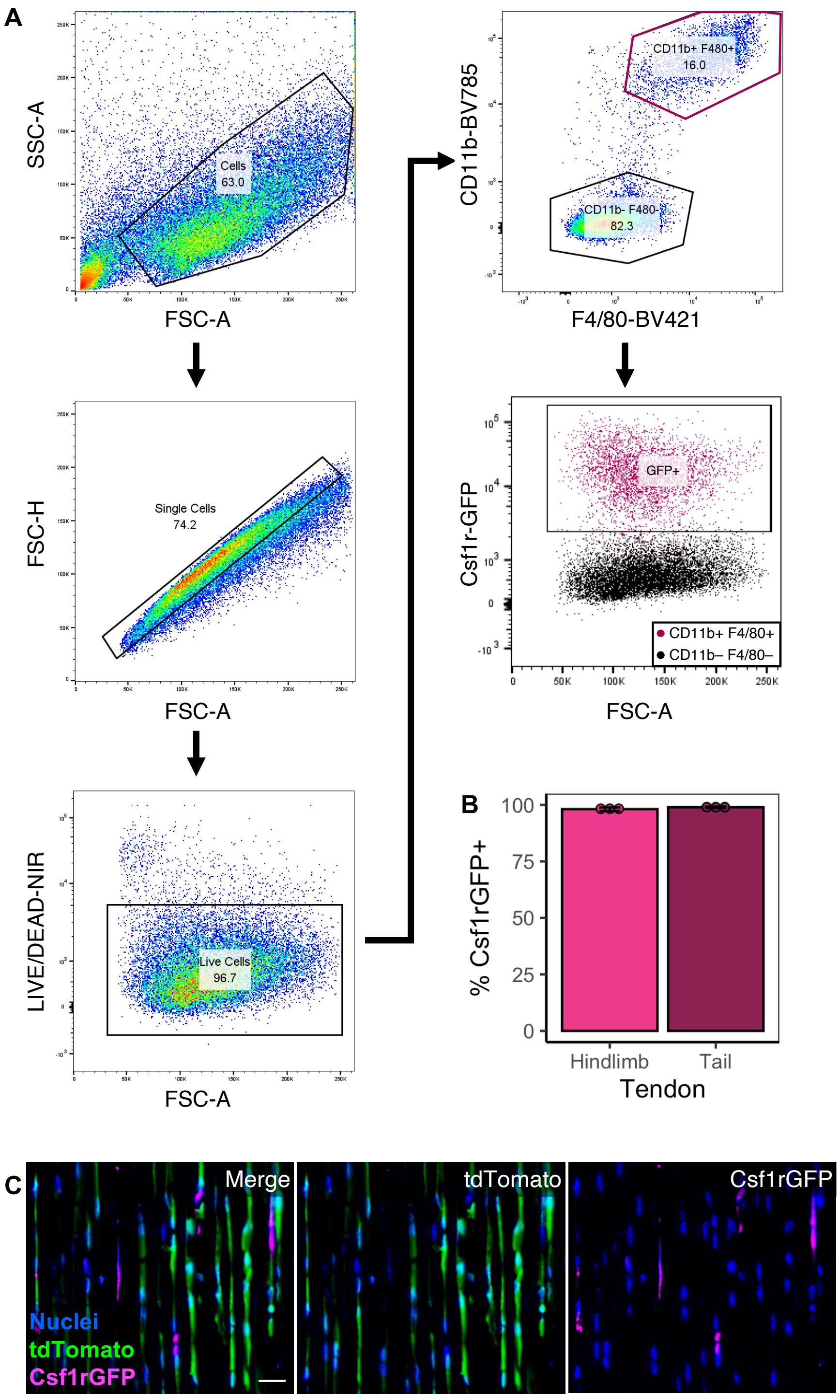

Supplementary Figure 2. Vast majority of CD11b$^+$ F4/80$^+$ and tdTomato$^-$ cells are Csf1rGFP$^+$ macrophages. **(A)** Representative gating strategy to subset CD11b$^+$ F4/80$^+$ cells (burgundy) from P28-P35 Csf1rGFP hindlimb and tail tendon cell suspensions. **(B)** Percentage of Csf1rGFP$^+$ cells within CD11b$^+$ F4/80$^+$ population in hindlimb and tail tendons. Data shown as mean ± SD (n = 3). **(C)** We generated Csf1rGFP;ScxCre;R26R-tdTomato transgenic reporter mice to label tenogenic lineage cells (tdTomato$^+$) and macrophages (Csf1rGFP$^+$) more robustly. Imaging of P28 ScxCre;Ai9;Csf1rGFP patellar tendon sections demonstrated that most tdTomato$^-$ cells are

Csf1rGFP$^+$. 99.4±0.8% of Csf1rGFP$^+$ cells were negative for tdTomato, confirming that resident macrophages are not derived from a tenogenic lineage (n = 2). 93.5±1.8% of all cells were tdTomato$^+$ and 5.1±1.3% of all cells were Csf1rGFP$^+$. Scale bar = 20 μm.

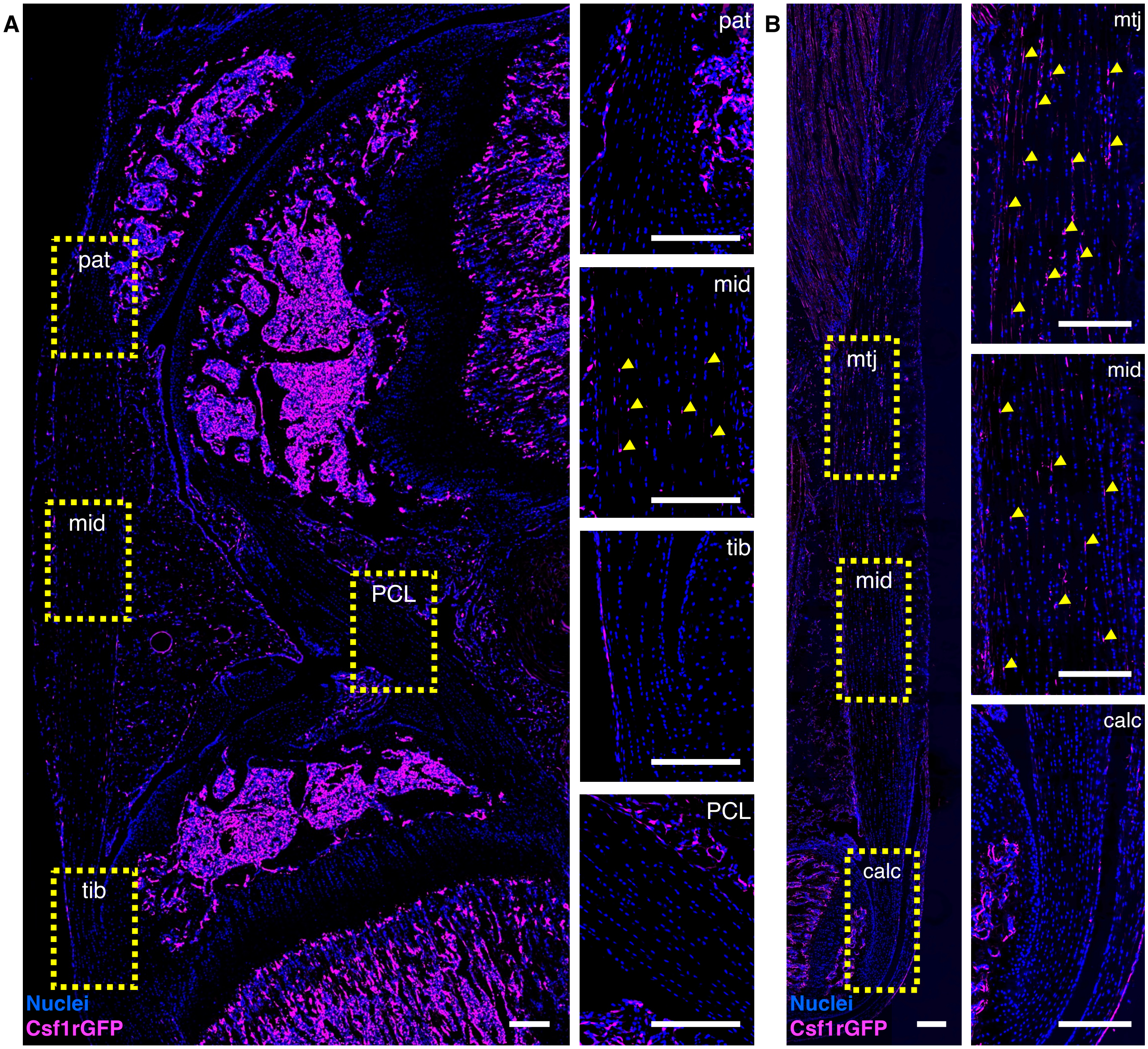

Supplementary Figure 3. Regional variation in macrophage density. **(A)** Representative image of P28 Csf1rGFP patellar tendon section with insets of the proximal tendon adjacent to the patella ("pat"), middle of the tendon ("mid"), distal tendon adjacent to the tibia ("tib"), and posterior cruciate ligament ("PCL"). Yellow arrowheads indicate Csf1rGFP^{+} cells within tendon fascicle. Scale bar = 200 µm. **(B)** Representative image of P28 Csf1rGFP Achilles tendon section with insets of the proximal tendon adjacent to the myotendinous junction ("mtj"), middle of the tendon ("mid"), and distal tendon adjacent to the calcaneus ("calc"). Yellow arrowheads indicate Csf1rGFP^{+} cells within tendon fascicle. Scale bar = 200 µm.

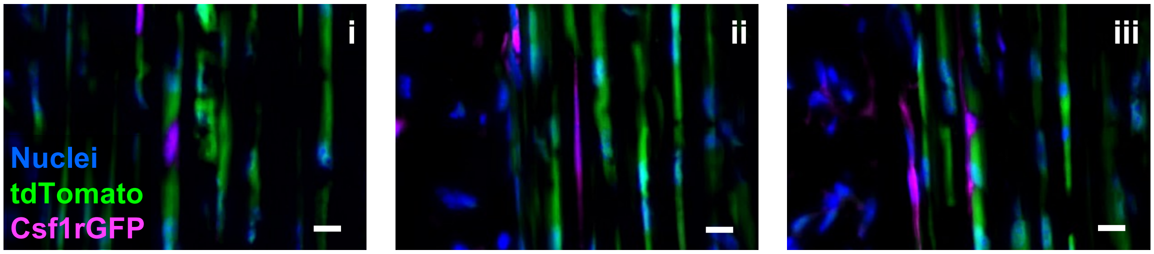

Supplementary Figure 4. Cellular morphology of tendon resident macrophages. Representative images of diverse morphologies of Csf1rGFP^{+} resident macrophages in P28 Csf1rGFP;ScxCre;Ai9 patellar tendon sections. Scale bar = 10 µm.

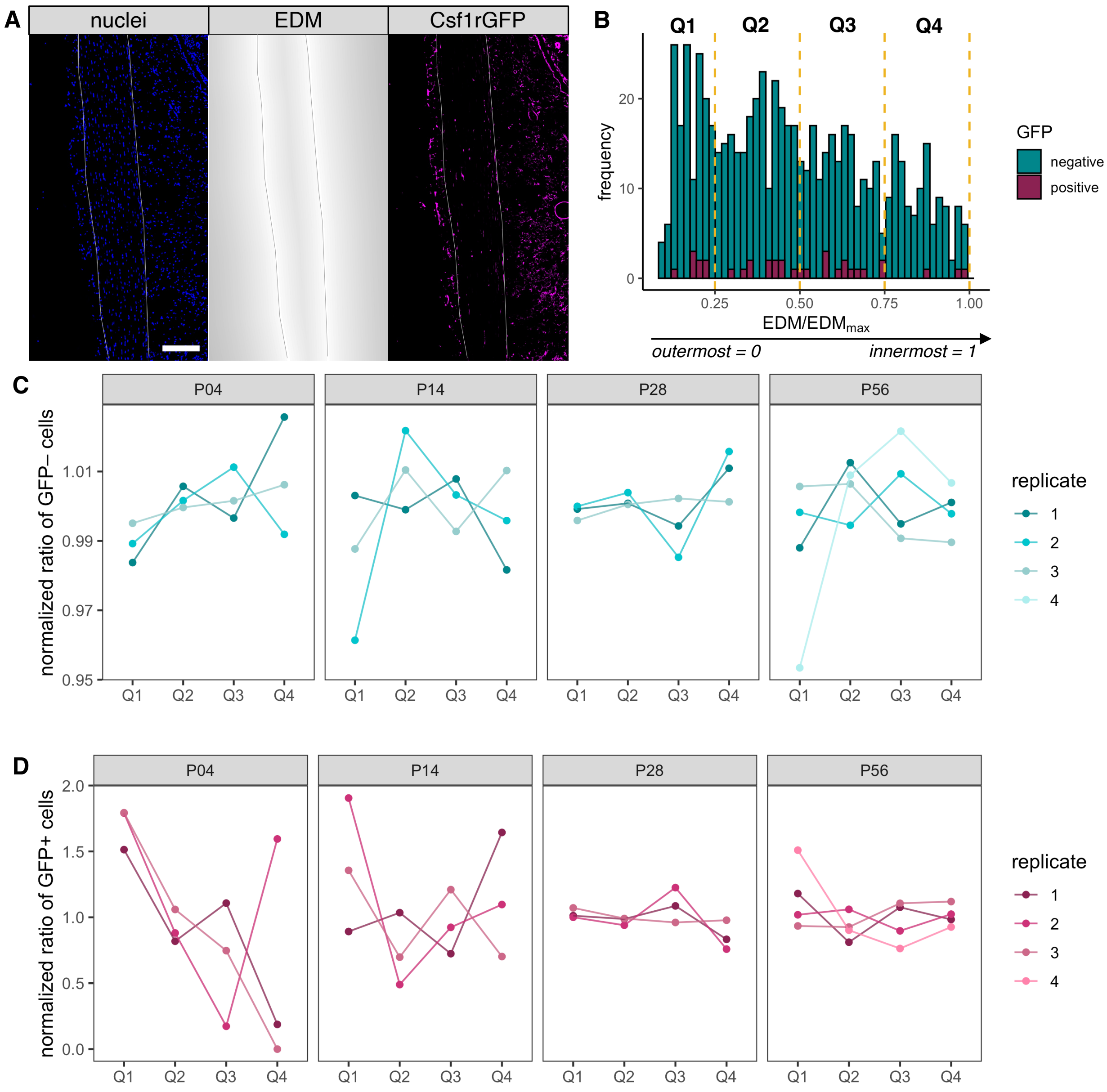

Supplementary Figure 5. Euclidean distance mapping (EDM) to determine distribution of macrophages across depth of tendon. **(A)** Representative images of nuclei, EDM, and Csf1rGFP channels. Anterior and posterior borders drawn in light gray. Contrast enhanced for better visualization of 16-bit EDM. Scale bar = 200 µm. **(B)** Example histogram of EDM intensity normalized by maximum EDM intensity (EDM_{max}) for one section with quartile limits indicated by yellow dotted lines. **(C)** Ratio of GFP^{-} cells to total cells in each quartile normalized by ratio of GFP^{-} cells to total cells in each section at P4, P14, P28, and P56 (n = 3-4), displayed for each individual biological replicate. **(D)** Ratio of GFP^{+} cells to total cells in each quartile normalized by ratio of GFP^{+} cells to total cells in whole section at P4, P14, P28, and P56 (n = 3-4). For all ages and for both populations, there were no significant differences in the normalized ratio across quartiles as determined by one-way ANOVA ($p > 0.05$).

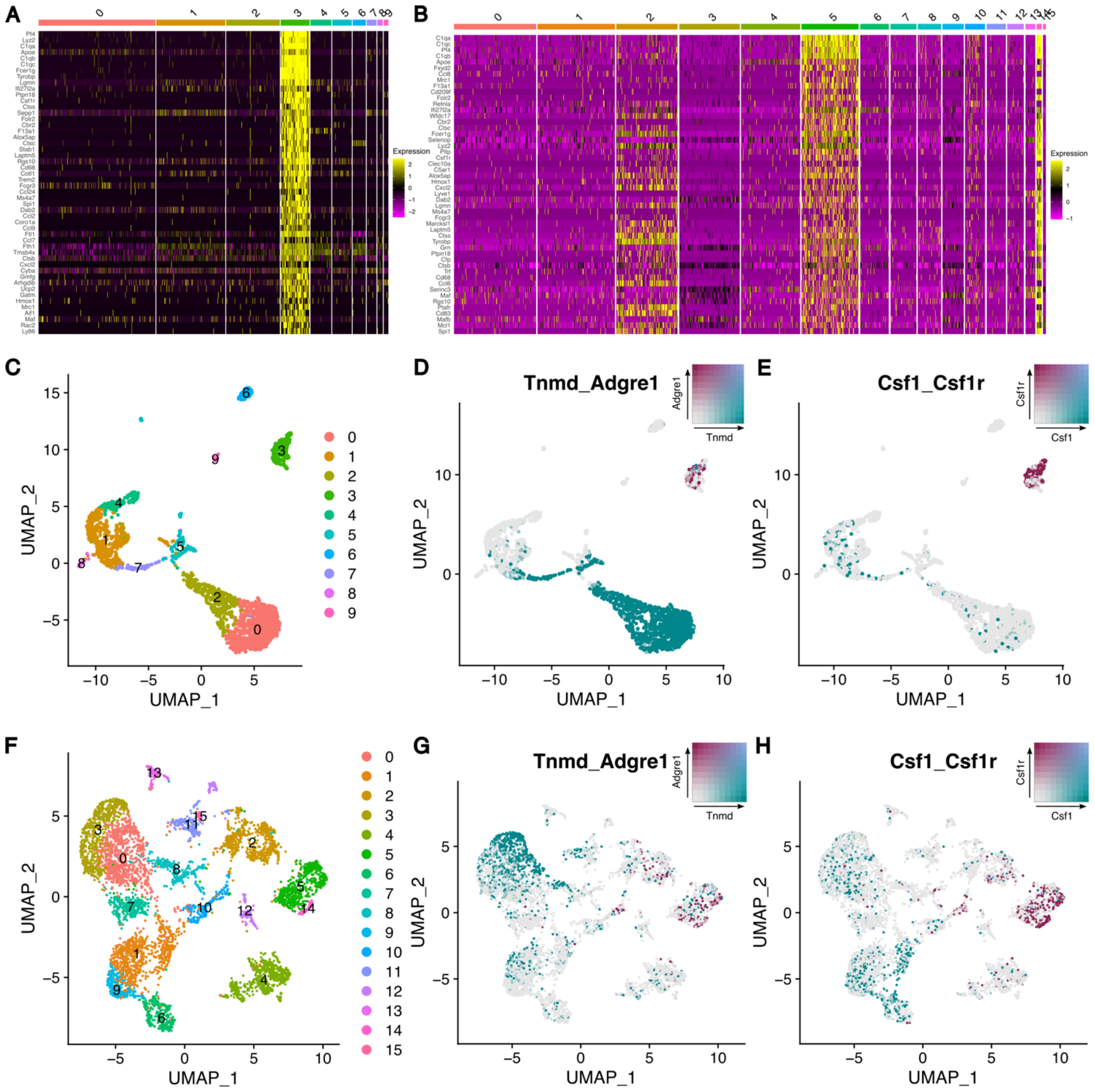

Supplementary Figure 6. Re-analysis of tendon single-cell RNA-sequencing (scRNA-seq) data. **(A)** Heatmap generated from re-analysis of P7 limb tendon scRNA-seq dataset (Tan et al., 2020), listing the top 50 markers that differentiate cluster 3, which is enriched in macrophage markers, from all other clusters. **(B)** Heatmap generated from re-analysis of 3-month-old patellar tendon scRNA-seq dataset (Harvey et al., 2019), listing the top 50 markers that differentiate cluster 5, which is enriched in macrophage markers, from all other clusters. **(C-E)** UMAPs generated from Tan et al. dataset. **(F-H)** UMAPs generated from Harvey et al. dataset. **(C & F)** UMAPs with cells colored by cluster assignments. **(D & G)** UMAPs showing the relative expression levels of tendon fibroblast marker *Tnmd* (teal) and macrophage marker *Adgre1* (burgundy). **(E & H)** UMAPs showing the relative expression levels of *Csf1* (teal) and *Csf1r* (burgundy). Note that, within the *Tnmd*$^{+}$ fibroblast clusters,

only a subset of cells expresses detectable levels of *Csf1*. *Csf1r* is exclusively expressed in the *Adgre1*$^+$ macrophage clusters.

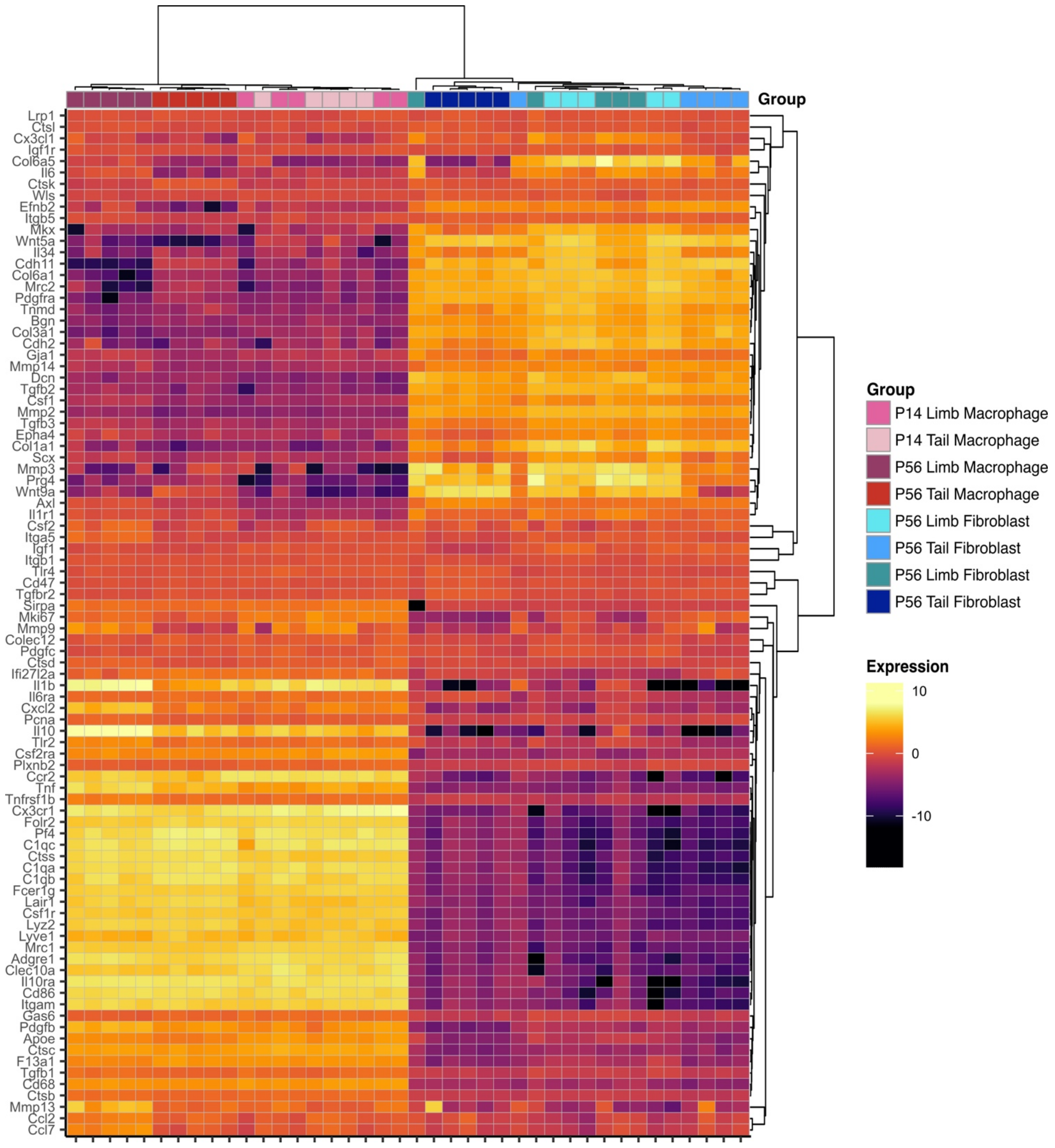

Supplementary Figure 7. Expression levels of all 90 genes (excluding 3 housekeeping genes and 3 technical outliers) and 40 samples from high-throughput qPCR study. Rows are centered; no scaling is applied to rows. Both rows and columns are clustered using correlation distance and average linkage.

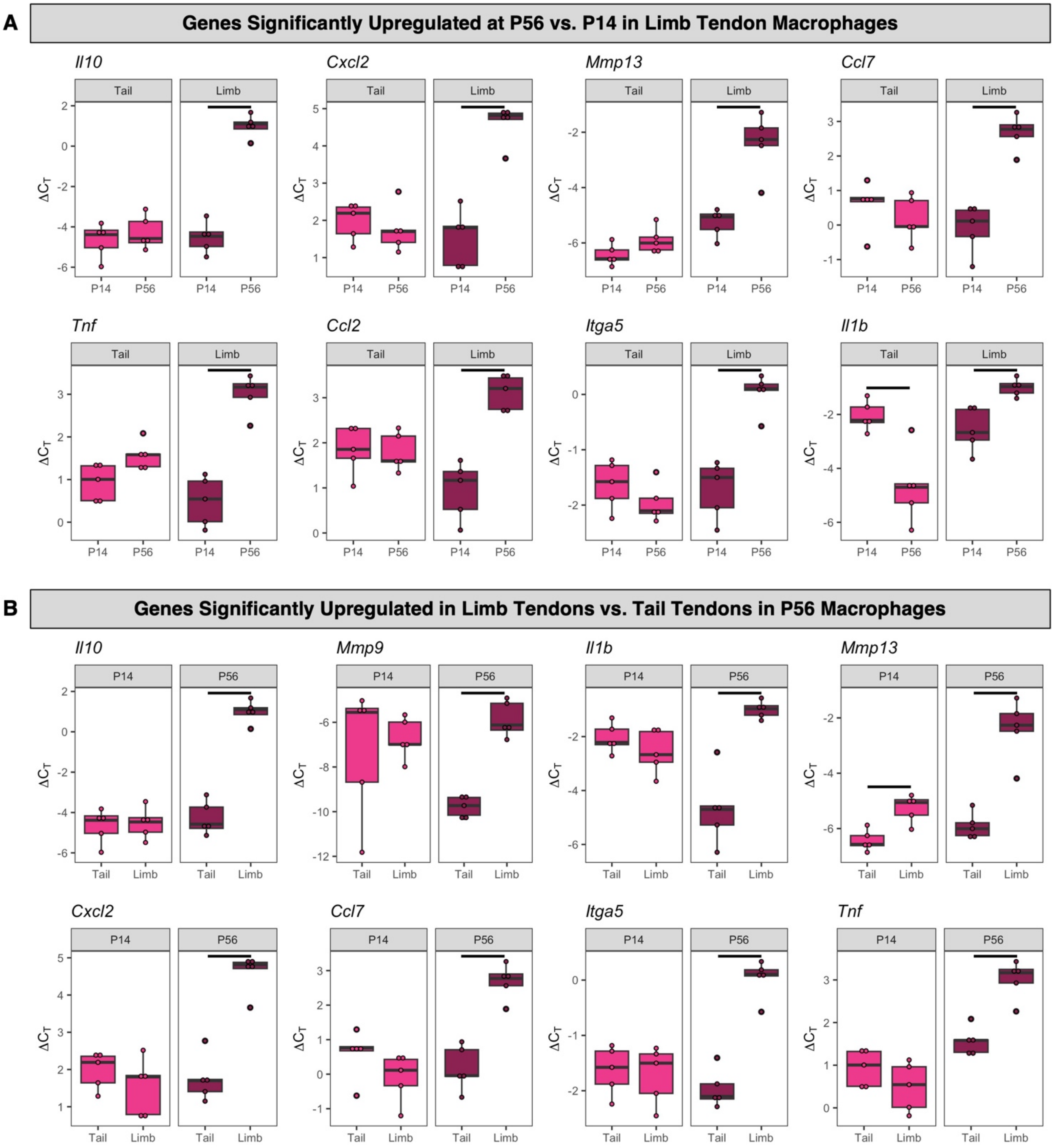

Supplementary Figure 8. (A) ΔC_T levels of genes significantly upregulated at P56 compared to P14 in limb tendon macrophages (n = 5). Average ΔC_T levels were not significantly different between P56 and P14 in tail tendon macrophages, except for *Il1b*. **(B)** ΔC_T levels of genes significantly upregulated in limb tendon compared to tail tendon macrophages at P56 (n = 5). Average ΔC_T levels were not significantly different between limb tendon and tail tendon macrophages at P14, except for *Mmp13*. Significance bars indicate $p < 0.05$ as determined by the Mann-Whitney U test.

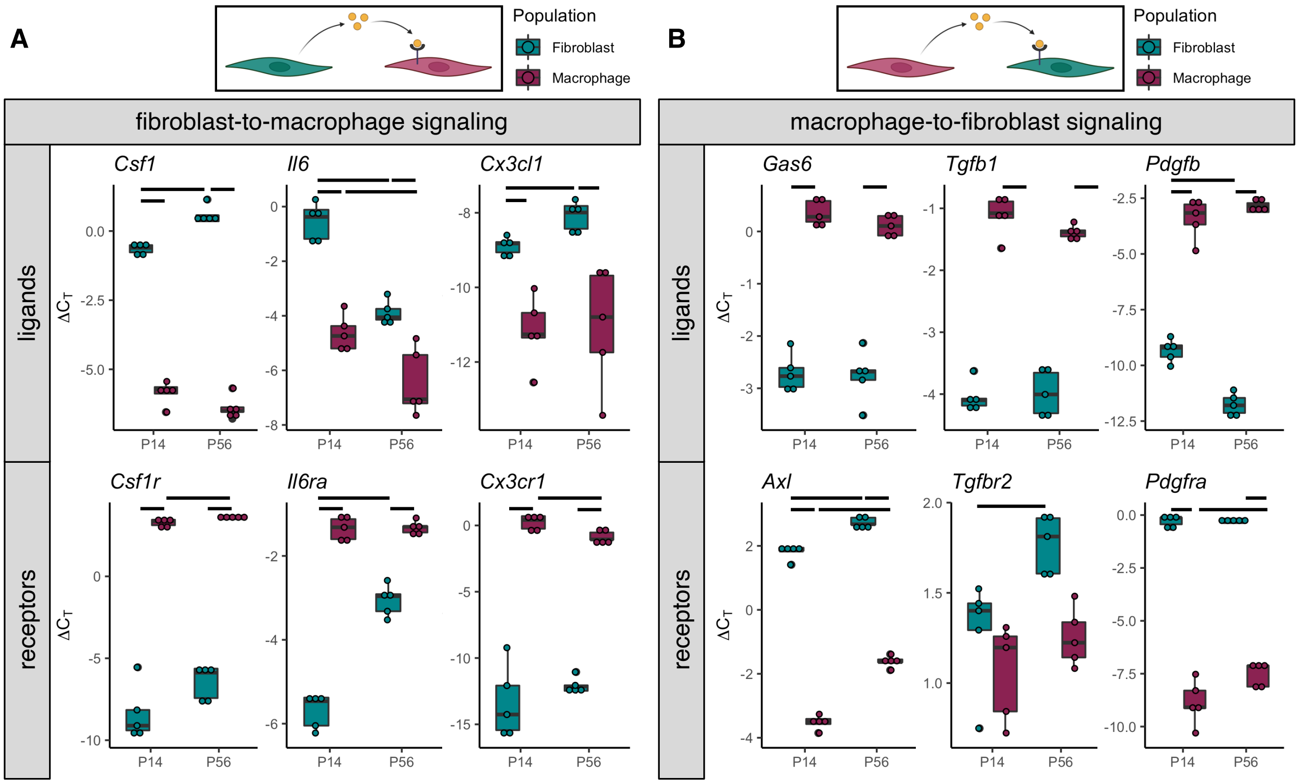

Supplementary Figure 9. Gene expression indicates potential tendon resident macrophage-fibroblast crosstalk in tail tendons. **(A)** ΔC_T levels of ligands enriched in tdTomato$^+$ fibroblasts (top) and receptors enriched in F4/80-Brilliant Violet 421$^+$ macrophages (bottom) from P14 and P56 tail tendons (n = 5). **(B)** ΔC_T levels of ligands enriched in F4/80-Brilliant Violet$^+$ macrophages (top) and receptors enriched in tdTomato$^+$ fibroblasts (bottom) from P14 and P56 tail tendons (n = 5). Significance bars indicate $p < 0.05$ as determined by the Mann-Whitney U test.

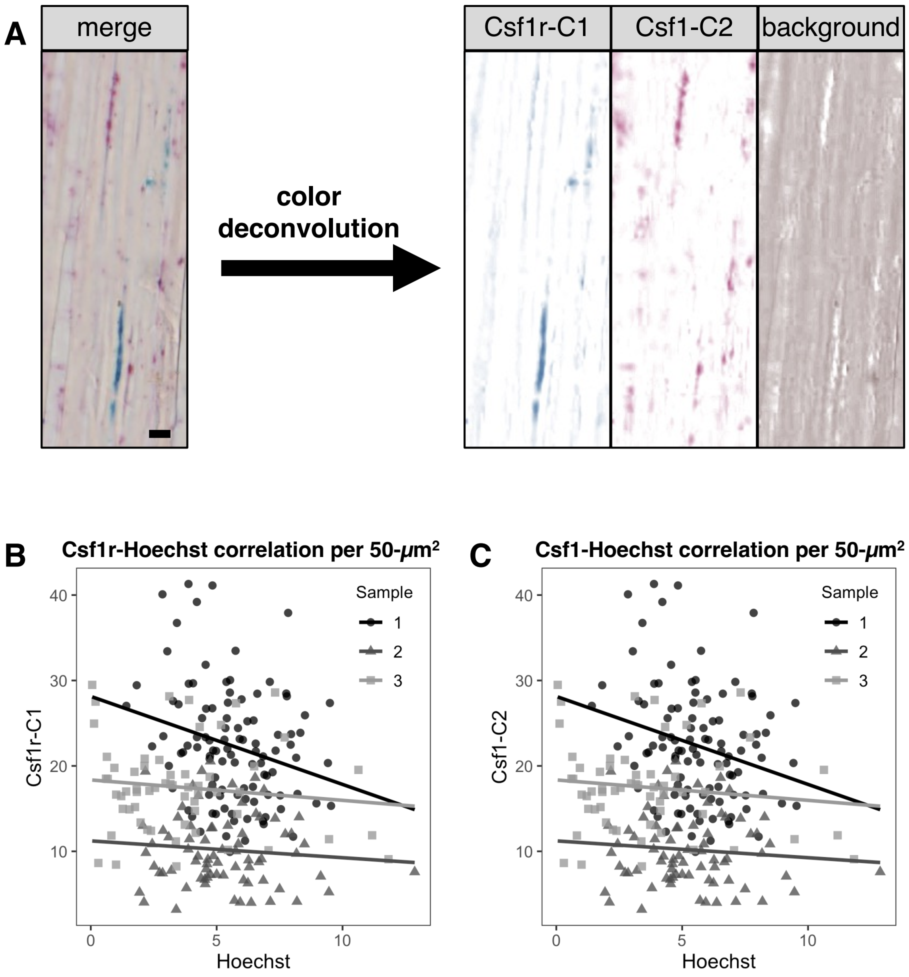

Supplementary Figure 10. (A) Example of color deconvolution of images of RNAScope ISH to obtain individual colors for Csf1r staining, Csf1 staining, and background. Scale bar = 10 μm. **(B)** Scatter plot showing average intensity of Csf1r-C1 and Hoechst staining. There was no significant correlation between Csf1r-C1 and Hoechst signals ($|\rho| < 0.3$ for all three samples). **(C)** Scatter plot showing average intensity of Csf1-C2 and Hoechst staining. There was no significant correlation between Csf1-C2 and Hoechst signals ($|\rho| < 0.3$ for all three samples).

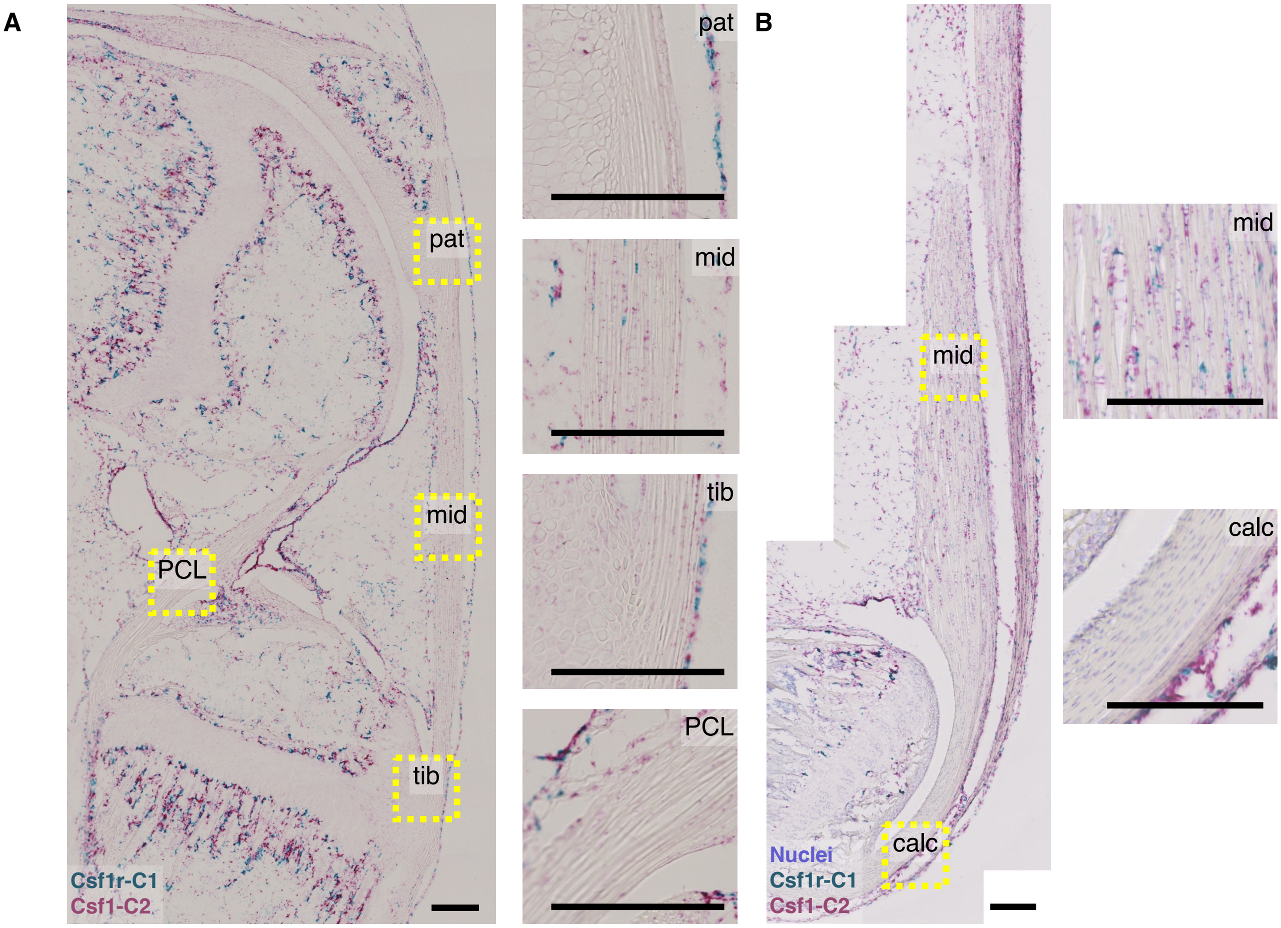

Supplementary Figure 11. Regional variation in *Csf1* expression. **(A)** Representative image of RNAScope ISH of *Csf1r* and *Csf1* on P28 knee sections with insets of the proximal tendon adjacent to the patella ("pat"), middle of the tendon ("mid"), distal tendon adjacent to the tibia ("tib"), and posterior cruciate ligament ("PCL"). Scale bar = 200 µm. **(B)** Representative image ISH of P28 ankle tendon sections with insets of the middle of the tendon ("mid") and distal tendon adjacent to the calcaneus ("calc"). Ankle sections were counterstained with hematoxylin. Scale bar = 200 µm. *Csf1* and *Csf1r* expression are lower in the tendon-to-bone attachment regions and in the PCL, where there are also fewer macrophages.

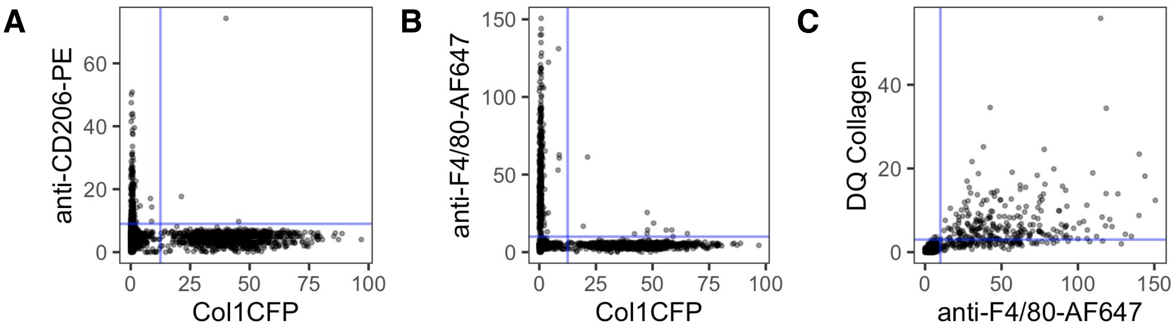

Supplementary Figure 12. Quantification of fluorescent signal intensity in cells isolated from tail tendon explants cultured with DQ Collagen. **(A)** Only 0.4% of Col1CFP+ cells were positive for CD206-PE. **(B)** Only 1.3% of Col1CFP+ cells were positive for F4/80-Alexa Fluor 647. **(C)** 67.9% of F4/80+ cells were positive for DQ Collagen.
